# Programmable DARPin-based receptors for the detection of thrombotic markers

**DOI:** 10.1038/s41589-022-01095-3

**Published:** 2022-08-08

**Authors:** Tobias Strittmatter, Yidan Wang, Adrian Bertschi, Leo Scheller, Patrick C. Freitag, Preetam Guha Ray, Pascal Stuecheli, Jonas V. Schaefer, Thomas Reinberg, Dimitrios Tsakiris, Andreas Plückthun, Haifeng Ye, Martin Fussenegger

**Affiliations:** 1 Department of Biosystems Science and Engineering, ETH Zurich, Basel, Switzerland; 2 Shanghai Key Laboratory of Regulatory Biology, Institute of Biomedical Sciences and School of Life Sciences, East China Normal University, Shanghai, People’s Republic of China; 3 Department of Biochemistry, University of Zurich, Zürich, Switzerland; 4 Johnson & Johnson, Allschwil, Switzerland; 5 Diagnostic Hematology, University Hospital Basel, Basel, Switzerland; 6 Faculty of Science, University of Basel, Basel, Switzerland; 7 Present Address: Roche Diagnostics, Penzberg, Germany; 8 Present Address: Laboratory of Protein Design & Immunoengineering, École Polytechnique Fédérale de Lausanne, Lausanne, Switzerland; 9 Present Address: Novartis Institutes for BioMedical Research, Basel, Switzerland

**Keywords:** Synthetic biology, Cardiovascular diseases, Protein design

## Abstract

Cellular therapies remain constrained by the limited availability of sensors for disease markers. Here we present an integrated target-to-receptor pipeline for constructing a customizable advanced modular bispecific extracellular receptor (AMBER) that combines our generalized extracellular molecule sensor (GEMS) system with a high-throughput platform for generating designed ankyrin repeat proteins (DARPins). For proof of concept, we chose human fibrin degradation products (FDPs) as markers with high clinical relevance and screened a DARPin library for FDP binders. We built AMBERs equipped with 19 different DARPins selected from 160 hits, and found 4 of them to be functional as heterodimers with a known single-chain variable fragments binder. Tandem receptors consisting of combinations of the validated DARPins are also functional. We demonstrate applications of these AMBER receptors in vitro and in vivo by constructing designer cell lines that detect pathological concentrations of FDPs and respond with the production of a reporter and a therapeutic anti-thrombotic protein.

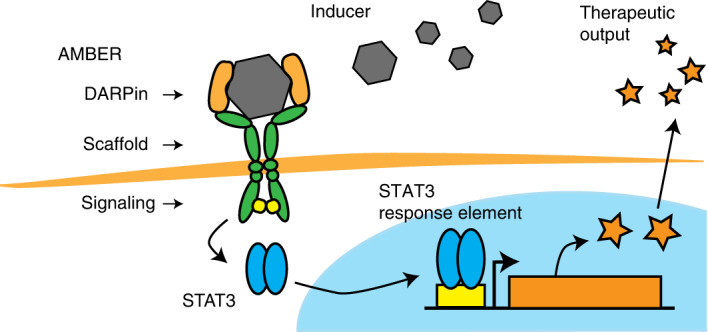

## Abstract (web summary)

Merging the generalized extracellular molecule sensor (GEMS) system with screening designed ankyrin repeat proteins (DARPins) identifies an advanced modular bispecific extracellular receptor (AMBER) for detection of fibrinogen degradation products.

## Main

Cell-based therapies are still hampered by the limited availability of cellular receptors for sensing clinical disease markers. To address this issue, various types of receptors have been employed to change cellular behavior^1^. These include well-known chimeric antigen receptors (CARs) that rely on endogenous signaling of T cells to drive T-cell responses to tumors. Other chimeric receptors that consist of fusions of extracellular receptor domains and intracellular signaling domains of native receptors have also been engineered to reroute cellular responses^2^. In addition, synthetic dimerization-dependent receptors can drive transgene expression by employing bacterial transcription factors^3^. However, all of these receptor systems require careful fine-tuning for each new ligand, and the ligand space is limited by the characteristics of the existing binding proteins.

So far, engineered receptors employed in mammalian synthetic biology have mostly utilized well-characterized protein–protein interactions of native receptor–ligand pairs or single-chain variable fragments (scFv) of antibodies. Moreover, although the ligand space for native receptor–ligand interactions is severely limited, protein scaffolds can be modified to recognize diverse targets. Indeed, some modified scaffolds have been used in high-throughput screenings for selecting binders to non-native targets completely unrelated to the preference of the original scaffold. Examples include fibronectin-III-derived monobodies^4^, αRep proteins containing HEAT-like repeats^5^, camelid-derived V_H_H domains, termed nanobodies^6^, and designed ankyrin repeat proteins (DARPins)^7,8^.

Various selection technologies have been used for antigen-binding antibody fragments and non-antibody scaffolds, including phage display^5^, yeast display^9^ and ribosome display^10^. The former two are advantageous for disulfide-containing proteins, while ribosome display can be used under reducing or oxidizing conditions and enables the screening of larger libraries, since no cells need to be transformed. Further, since PCR amplification is an integral part of the selection cycle in ribosome display, it is straightforward to introduce error-prone PCR to increase sequence variation and allow for affinity maturation^11^. Ribosome display is particularly suitable for proteins that fold well during cell-free translation, such as DARPins, which are small binding proteins of 14–17 kDa^7,12^. Notably, DARPins add value by expanding the synthetic receptor landscape, because their epitope preference is different from that of antibody-like binders such as scFvs^13,14^.

**Fig. 1:**
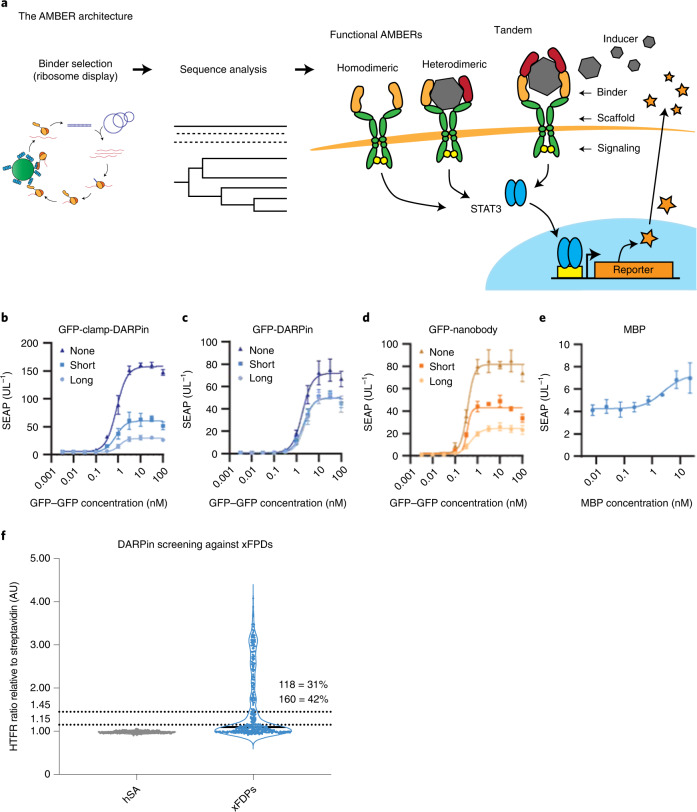
AMBERs are generated using ribosome display and can function in different configurations to detect soluble targets such as GFP or MBP. **a**, Activation of the sensor triggers the signal transducer and activator of transcription 3 (STAT3) transcription factor pathway, which is rerouted towards expression of a therapeutic output (TNK or hirudin, Hir) or a reporter protein (SEAP or NLuc). A four-step pipeline for the generation of functional AMBERs. First, binders (DARPins) are selected by ribosome display and the genetic information of candidate binders is analyzed. Next, genetically distinct binders are used to build AMBERs, which are tested in cellular assays in homodimeric or heterodimeric configuration. AMBERs on the cell surface can be employed to detect fibrin-derived proteins via engineered extracellular binding domains. After the identification of functional binders, they can be used to build more compact tandem receptors with similar specificity. **b**–**d**, Spatial requirements of AMBERs were tested by inducing AMBERs specific for GFP on the basis of a published clamp-DARPin (**b**), a corresponding single DARPin (**c**) and a nanobody in a homodimeric receptor configuration (**d**). GFP–GFP fusion proteins having a long GGGGS_4_ (20 amino acids), short GGGGS_1_ (5 amino acids) or no linker between the GFP domains were used as analytes. **e**, An additional AMBER was designed for the detection of bacterial MBP. Its EC_50_ value was estimated to be 0.85 nM. **f**, Results from HTRF assay depicted as the ratio of maximum fluorescence of the sample versus the streptavidin control. Streptavidin is used to couple biotinylated target protein to beads during the selection run. DARPins are labeled via a FLAG tag with an antibody coupled to a fluorescent acceptor dye (d2) while xFDPs are labeled in a similar fashion via their biotin tag with streptavidin carrying a Tb FRET donor. Binding of a DARPin to FDP protein hence results in an increase of FRET efficiency (increased signal-to-noise ratio). AU, arbitrary units. EC_50_ values of clamp-DARPin-based AMBER (**b**) were 1.14 nM (long linker), 0.77 nM (short linker), and 0.84 nM (no linker) with 95% confidence intervals (CIs) of [0.91 to 1.46], [0.52 to 1.09], and [0.73 to 0.96]; the single DARPin-based AMBER (**c**) showed EC_50_ values of 2.27 nM (long linker), 2.00 nM (short linker), and 1.83 nM (no linker) with 95% CIs [1.83 to 2.77], [1.64 to 2.44], and [1.48 to 2.27]; and the nanobody-based AMBER (**d**) showed EC_50_ values of 0.49 nM (long linker), 0.34 nM (short linker), and 0.35 nM (no linker) with 95% CIs [0.38 to 0.63], [0.29 to 0.43], and [0.30 to 0.41]. Data are presented as mean ± s.d. of *n* = 3 independent samples. Source Data

We recently developed the generalized extracellular molecular sensor (GEMS) platform, consisting of an extracellular target-binding moiety linked to the erythropoietin receptor (EpoR) extracellular domain, for the generation of designer receptors^15^. GEMS receptors are activated if each receptor chain binds to the ligand independently in a sandwich conformation, with the ligand bridging the two receptor chains (Fig. 1a). Though the GEMS platform enables modular swapping of binding domains and signal transduction domains, changing the receptor specificity still relies on the availability of a suitable target-binding domain. Therefore, we set out to combine the GEMS platform with high-throughput binder-screening technology to generate a customizable advanced modular bispecific extracellular receptor (AMBER). While the GEMS platform capitalizes on a limited set of reported antibody-like binders such as scFvs, the AMBER system has been tailored for DARPins obtained by high-throughput screening. To validate our design strategy, we benchmarked DARPin-based AMBERs against AMBERs constructed with nanobodies in a GFP-receptor context. We further demonstrated the generalizability of our design by using reported binders to build an AMBER for the detection of bacterial maltose-binding protein (MBP). Then, we characterized the full AMBER pipeline in more detail by generating binders for human fibrin degradation products (FDPs) to build AMBERs that would serve as cellular sensors for blood coagulation events. FDPs are already used as a biomarker to rule out life-threatening pathologic coagulation events^16,17^. We also demonstrated the translational potential of the AMBER system by engineering mammalian cells equipped with AMBERs to detect pathological coagulation events and to respond by triggering the secretion of a therapeutic protein, either tenecteplase (TNK) or the anti-coagulant hirudin.

Overall, our results suggest that the AMBER pipeline provides streamlined and predictable methodology for the generation of new cellular receptors for soluble targets of choice. We believe this technology will facilitate research on cellular signaling, as well as the development of future cellular therapies to treat diseases for which no relevant cellular receptors are yet known.

## Results

### Design requirements of AMBERs

We combined the AMBER system (Fig. 1a) with established high-throughput DARPin selection technology to design a four-step pipeline for generating functional AMBERs (Fig. 1a). In brief, DARPins are selected by ribosome display and their sequences are classified by phylogenetic comparison and pairwise amino acid alignments. Distinct DARPins are fused to the receptor scaffold and tested for induction in a homodimeric or heterodimeric receptor setup. Finally, we developed a more compact tandem receptor design featuring two binders on a single receptor, thereby reducing the amount of genetic material required for transfection. This design may be applied to validated DARPin binders.

In the AMBER platform, receptor activation relies on binding of both receptor chains to their target, inducing receptor dimerization. From the crystal structure of activated EpoR (Protein Data Bank (PDB) ID: 1EER), we estimated the optimal distance between the attachment sites of the C termini of the two binders (N termini of the EpoR chains; orange in Fig. 1a) to the extracellular domains (green in Fig. 1a) to be less than 8 nm. However, larger structures may also be recognized^18^.

To characterize the spatial requirements of the receptor system and to benchmark the performance of DARPins in this context, we built AMBERs equipped with either a clamp-DARPin, consisting of two DARPins forming a high-affinity binder^19^, a single DARPin derived from that clamp-DARPin binder^19^ or a nanobody binder^20^, each targeting GFP. We transfected HEK-293T cells with these receptors in a homodimeric configuration and induced them with purified GFP–GFP fusion proteins having a long and flexible GGGGS_4_ linker, a short GGGGS_1_ linker, or no linker at all to allow for differently spaced epitopes (Fig. 1b–d, Supplementary Fig. 1a and Supplementary Table 3). Nanomolar half maximal effective concentration (EC_50_) values were obtained, and GFP–GFP without any linker showed the strongest activation (Fig. 1b–d). A clear effect of linker length on EC_50_ was observed for clamp-DARPin-based AMBER and nanobody-based AMBER. We also confirmed the functionality of AMBER with scFv/DARPin binder combinations by designing and testing a receptor for bacterial MBP, using a published scFv sequence (PDB ID: 7JTR chain B) together with a DARPin (PDB ID: 1SVX) fused through a stiff (EAAAK)_4_ linker. Its estimated EC_50_ was 0.85 nM (Fig. 1e).

To evaluate the AMBER platform for detection of disease markers, we next established functional receptors for D-dimers, which contain identical epitopes along the whole 45 nm length of the dimeric fibrinogen molecule^21,22^, providing a range of differently spaced epitope pairs.

### DARPin selection

**Extended Data Fig. 1:**
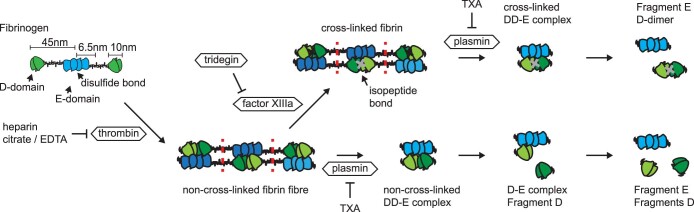
Overview of processes involved in fibrinolysis. Overview of processes involved in fibrinolysis. Fibrinogen forms a head-to-head dimer via the E-domain (blue) that is covalently linked via disulfide bonds. Upon activation with thrombin, fibrinogen oligomerizes via interactions of its D-domains (green) with the E-domain to form an insoluble non-cross-linked fibrin mesh. The mesh is stabilized by factor XIIIa, which introduces isopeptide bonds between adjacent D-domains. Fibrinolysis is mediated by plasmin, which cleaves fibrin into smaller soluble fragments. Depending on whether the fibrin was cross-linked prior to plasmin digestion, different types of fragments arise. Degradation products include fragment E comprising the E-domain of fibrinogen (blue), fragment D (green) and cross-linked D-dimers (green, indicated by orange crosses), as well as complexes thereof. Many of these products contain the same epitopes.

Multiple intermediates and derivatives of fibrin, which partially share the same epitopes, are formed during coagulation and fibrinolysis (Extended Data Fig. 1). To validate the AMBER platform we chose commercially available cross-linked fibrin degradation products (xFDPs) enriched in D-dimers (fibrin D-fragments linked by an isopeptide bond, compare Extended Data Fig. 1) as a target for FDP binder selection because of their clinical relevance^16^. This preparation also contains traces of D-E complex (120 kDa) and DD-E complex (220 kDa) with large surface areas that should present numerous epitopes (Supplementary Fig. 1b).

**Extended Data Fig. 2:**
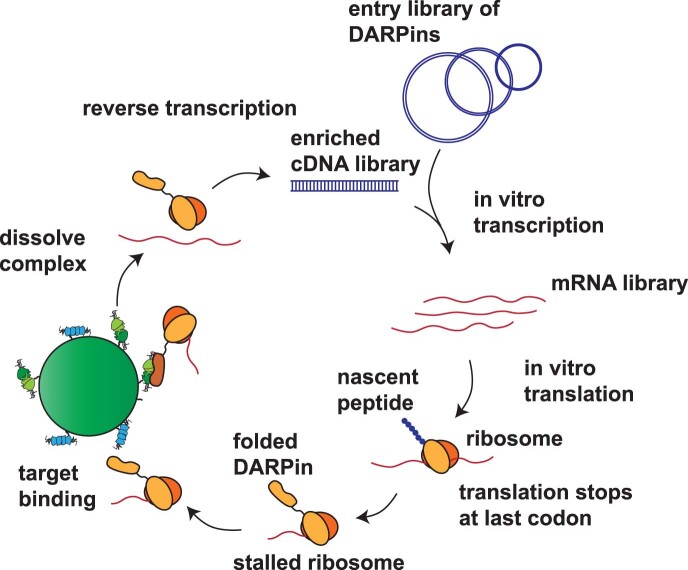
Ribosome-display-based DARPin selection. Overview of ribosome-display-based DARPin selection to generate novel binding domains. An optimized DARPin library containing a mixture of N3C-DARPins with randomized and non-randomized N- and C-caps is transcribed to mRNA, which is translated in vitro. Since the mRNA lacks a stop codon, the ribosome is stalled on the mRNA with the nascent peptide still attached via a fused tether upon completion of translation. The fully folded DARPin can now bind to FDPs coupled to streptavidin-coated beads via their biotinylated lysine residues. FDP-DARPin-ribosome-mRNA complexes are washed and subsequently dissolved. The resulting mRNA is reverse-transcribed to yield an enriched plasmid library, which is subjected to three additional rounds of the same selection cycle. The products of the final round are transformed into *E. coli* for single clone screening.

We biotinylated the xFDP preparation and applied four rounds of in vitro ribosome display-based screening to generate DARPins (Methods; Extended Data Fig. 2). We then screened 380 DARPins for target binding by homogeneous time-resolved fluorescence (HTRF)-based assay (Fig. 1f). We verified the absence of unspecific binding to human serum albumin (hSA), present as a stabilizer in the xFDP preparation.

Of the 380 DARPins, 160 (42%) gave a fold induction >1.15 (a value chosen on the basis of our previous screening experience), and of these, 118 (31%) were considered good binders with a fold induction >1.45. To cover a broad range of epitopes, we selected 24 high-affinity and 8 medium-affinity DARPins for Sanger sequencing. Among them, 19 DARPins had unique sequences, though some showed high sequence similarity (Supplementary Fig. 2), and 15 (79%) were monomeric (Supplementary Fig. 3). We used all 19 binders in subsequent receptor designs, as constraints introduced by receptor fusion might affect dimerization in the final receptor constructs.

### Homodimeric, heterodimeric, and tandem AMBER configurations

**Fig. 2:**
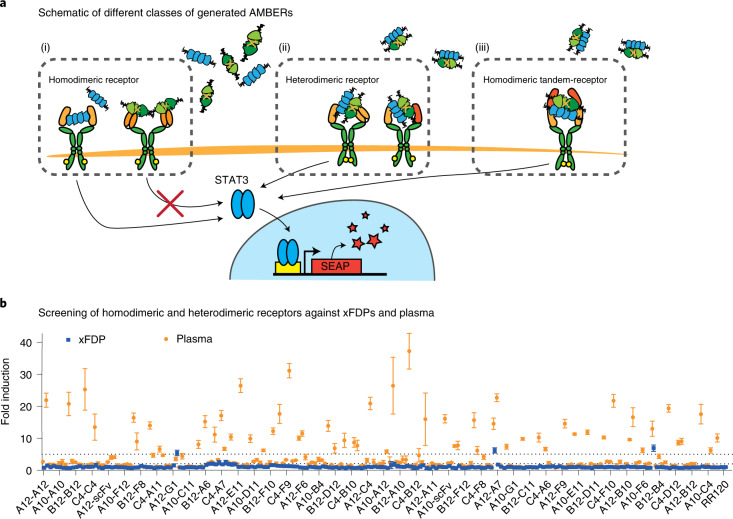
AMBERs bind different targets depending on receptor configuration and inducer composition. **a**, Engineered receptors on the basis of the GEMS platform bind to fibrin degradation products, including fragment E (blue), fragment D (green) and cross-linked D-dimers (green, indicated by orange crosses), as well as complexes thereof. Binding induces STAT3-mediated gene expression of therapeutic proteins or the reporter protein human SEAP. (i) Homodimeric receptors present one binding moiety only and rely on symmetrically presented epitopes on the target. (ii) Heterodimeric receptors display two different binding moieties, similarly to (iii) homodimeric tandem receptors, which comprise two binders that are fused on top of the receptor chains in a head-to-tail fashion. **b**, Combinations of different receptors comprising different binding domains were tested for their response to purified xFDP protein (blue dots) as well as plasma (orange dots). Values are normalized to uninduced samples of the same receptors and represent mean ± s.d. of *n* = 3 independent samples. Positive responses were divided into two categories: weak (2 < fold induction < 5 versus uninduced samples) and strong (fold induction > 5). Source Data

Different receptor configurations were explored to find the best setup (Fig. 2a). A homodimeric receptor configuration in which all receptors have the same binding module is the smallest and most straightforward receptor architecture (Fig. 2a). However, homodimeric receptors rely on the presence of two identical epitopes close together, so functional receptors require dimeric or symmetric ligands.

Heterodimeric receptors are pairwise combinations of receptors with different binding moieties (Fig. 2a), and thus require two independent receptor chains. Pairwise combinations grow exponentially with the number of available binders (*N* pairwise combinations of two out of *n* elements: *N* = 0.5*n*^2^ − 0.5*n*), greatly increasing the likelihood of finding active receptors.

Tandem receptors consist of two binders fused in sequence to the N terminus of the receptor scaffold, resulting in increased avidity and possibly higher sensitivity (Fig. 2a). They require a smaller genetic payload than heterodimeric receptors (2.5–2.9 kb for the receptor alone), allowing them to be used in size-restricted vectors, such as recombinant adeno-associated viruses (rAAVs)^23^. However, the combinatorial space when all binders are tested in all possible configurations in heterodimeric tandem receptors increases to the power of four for each binder.

For AMBER, we therefore decided to screen homodimeric or heterodimeric receptors before confirming the functionality of tandem receptors built with the validated binders.

### AMBER screening setup and specificity controls

To benchmark new FDP-binding DARPins and to explore cross-platform binder combinations, we used a single-chain variable fragment (scFv) targeting human D-dimers^24^. We equipped the AMBER platform with the anti-D-dimer-scFv (scFv-AMBER) and the 19 FDP-binding DARPins, obtaining 20 receptors, each bearing a single binding moiety on the extracellular domain. HEK-293T cells were co-transfected with plasmids, each encoding a single receptor under the control of a constitutive synthetic promoter derived from human cytomegalovirus (hCMV) (P_hCMV_-receptor-pA), along with a plasmid encoding a constitutively expressed STAT3 transcription factor (pLS15, P_hCMV_-STAT3-pA) and a secreted human placental alkaline phosphatase (SEAP) reporter plasmid controlled by two STAT3 response elements (RE) and a minimal version of P_hCMV_ (pLS13, RE2-P_hCMVmin_-SEAP-pA).

Insensitivity of the receptors to erythropoietin (EPO) was confirmed by the absence of EPO-mediated activation (Supplementary Fig. 4a). A construct based on an scFv responding exclusively to the non-toxic industrial dye Reactive Red (RR120)^15^ was used as a negative control for testing potential receptor-independent effects (Supplementary Fig. 4b). We assessed the impact of purified human xFDPs on the productivity and viability of HEK-293T cells by evaluating constitutive SEAP expression (Supplementary Fig. 4c) and by resazurin-based assay (Supplementary Fig. 4d), respectively. xFDPs were non-toxic up to 10 µg ml^−1^. Human plasma at concentrations up to 2% (v/v) had little effect on constitutive SEAP expression (Supplementary Fig. 4e) or viability (Supplementary Fig. 4f).

### AMBER screening

**Extended Data Fig. 3:**
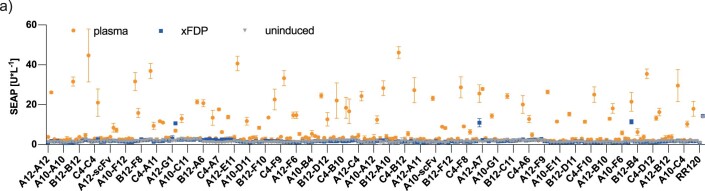
Screening of homodimeric receptors in HEK-293T. Screening of homodimeric receptors in HEK-293T. Cells were incubated with 0.5 µg/mL xFDPs or 0.5 % (v/v) reconstituted human plasma in complete DMEM containing 10 % FCS for 24 h, and SEAP reporter activity was measured. RR120 was included as a negative control and constitutive expression of SEAP was used as a reference. All values are means ± SD of n = 3 independent samples. Source Data

We finally screened all 210 possible pairwise combinations of receptors (including all 20 homodimers) (Extended Data Fig. 3 and Supplementary Fig. 5). Induction with xFDPs yielded four weak (2 < fold induction < 5; 1.9%) and three strong (fold induction > 5; 1.4%) receptors (Fig. 2b). Notably, all receptors responding strongly to xFDPs were heterodimers formed by co-expression of a DARPin with the scFv receptor, indicating the importance of synergies among binders covering different epitopes for the generation of functional receptors.

Human citrate-treated plasma was tested to evaluate the system performance in a more native setting and to investigate responses to FDP species underrepresented in the DARPin selection and screen. We found weak responses for 37 (17.6%) and strong responses for 49 (23%) receptor combinations of which 77% included DARPin A6, A7, B4 or F6. We found no correlation of HTRF signal intensity from DARPin selection with the number of functional receptors or the receptor signal strength in a homodimeric setup (Supplementary Fig. 6). This was not unexpected, since HTRF signal strength depends on both affinity and epitope location. Interestingly, receptor activity in a homodimeric setting seems predictive for the number of functional receptor combinations with fold induction > 2. Some functional receptors contained DARPins that showed oligomerization behavior (for example, F9-DARPin; Supplementary Fig. 3), indicating that auto-dimerization of the binder alone does not necessarily limit its use in a receptor context.

### Characterization of candidate AMBERs

**Fig. 3:**
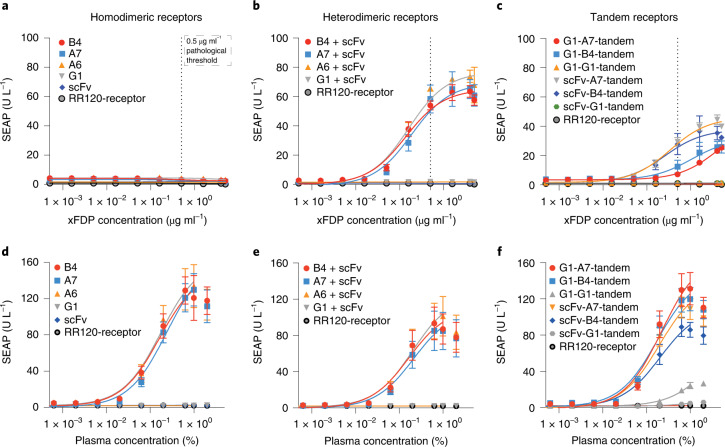
Measurement of AMBER affinities to xFDP and plasma. **a**–**c**, Functional AMBER systems for the detection of (x)FDP started to respond well below the clinically relevant concentration of 0.5 µg ml^−1^ (dotted line). **d**–**f**, When tested for reactivity against human plasma the sensitivity of the system increased remarkably. **a**–**c**, Solid lines are non-linear fits. Values for the highest concentration were excluded from the fit in **d**–**f** to improve the fits for lower-to-medium concentrations. The RR120 receptor serves as a negative control. The *x*-axis is log-scale and all values are mean ± s.e.m. of *N* = 3 independent experiments of *n* = 3 samples. Source Data

We next characterized DARPins A6, A7, B4, and less-active DARPin G1, as well as the scFv, in a homodimeric receptor configuration. Reporter levels showed little xFDP concentration dependence for receptors based on A6, A7 and B4 DARPins (Fig. 3a). A possible explanation is binding of large antigens to both receptor chains, thereby reducing basal (‘leaky’) activity by stabilizing pre-formed receptor dimers in an inactive conformation. Alternatively, the epitopes in a single xFDP molecule might be oriented such that they cannot be simultaneously engaged by both binders.

By contrast, we recorded ON-type switching behavior with up to 110-fold induction in response to human plasma for the same DARPins, but not for the scFv (Fig. 3d). This may indicate that these DARPin receptors are activated by another protein that is not present in the xFDP preparation (compare Supplementary Fig. 1b). Plasma contains fibrinogen, a precursor of (x)FDPs that is activated by thrombin contained in the medium’s fetal calf serum (FCS) supplement, leading to multiple possible fibrinogen-derived inducer molecules (Extended Data Fig. 1). We performed further experiments to identify the responsible molecule (see below). Here, we first focus on the receptor configurations and binder characteristics.

We examined the four candidate DARPins in combination with the scFv as heterodimeric receptor pairs. In contrast to the homodimeric configuration, these receptors dose dependently respond to xFDPs with inductions of up to 25-fold and EC_50_ values of 0.15, 0.19, and 0.12 µg ml^−1^ for DARPins A6, A7, and B4, respectively (Fig. 3b). These EC_50_ values are below the pathological threshold for D-dimer of 0.5 µg ml^−1^ in blood^25^. Induction with plasma yielded similar dose–response relationships to those in the homodimeric configuration (Fig. 3e). However, the heterodimeric setup also allows for homodimeric receptors at the cell surface, and the latter most likely dominate the plasma induction response. Nevertheless, only heterodimeric receptors were also activated by xFDPs. Thus, it might be possible to distinguish different stages or states of coagulation by using different receptors (compare Extended Data Fig. 1).

### Generating tandem AMBERs

After identifying functional DARPin binders and scFv combinations in the homodimeric and heterodimeric screens, we fused all 16 combinations of tested DARPins A7, B4, G1, and the scFv to the receptor scaffold in tandem orientation (Fig. 2a). Three (19%) receptors, all containing a DARPin in combination with the scFv, were sensitive to xFDPs (Supplementary Fig. 7). All but one of the DARPin-based receptors and all but two of the scFv–DARPin combinations were plasma responsive. Notably, we found a working receptor for each DARPin successfully tested before (Supplementary Fig. 7a,b). The scFv alone did not respond to either plasma or xFPDs in tandem configuration, in accordance with the results for the homodimeric receptors.

The DARPin-only tandem receptors detected xFDPs dose dependently, albeit with lower sensitivity than heterodimeric scFv combinations (EC_50_ 4.3 µg ml^−1^ for G1–A7, 1.2 µg ml^−1^ for G1–B4; Fig. 3c). Tandem receptors comprising DARPins and the scFv were as sensitive as their heterodimeric counterparts; for example, EC_50_ 0.31 µg ml^−1^ (versus 0.19 µg ml^−1^) for the scFv–A7 receptor and 0.25 µg ml^−1^ (versus 0.12 µg ml^−1^) for the scFv–B4 receptor. Induction with plasma led to increased signal intensities for all tandem receptors, even the previously non-responding G1–G1 (Fig. 3f). Thus, compact tandem receptors exhibit full functionality and mostly reflect the features of the corresponding heterodimeric receptors. The question of whether tandem configurations also allow for different ligands to be detected is addressed below.

**Extended Data Fig. 4:**
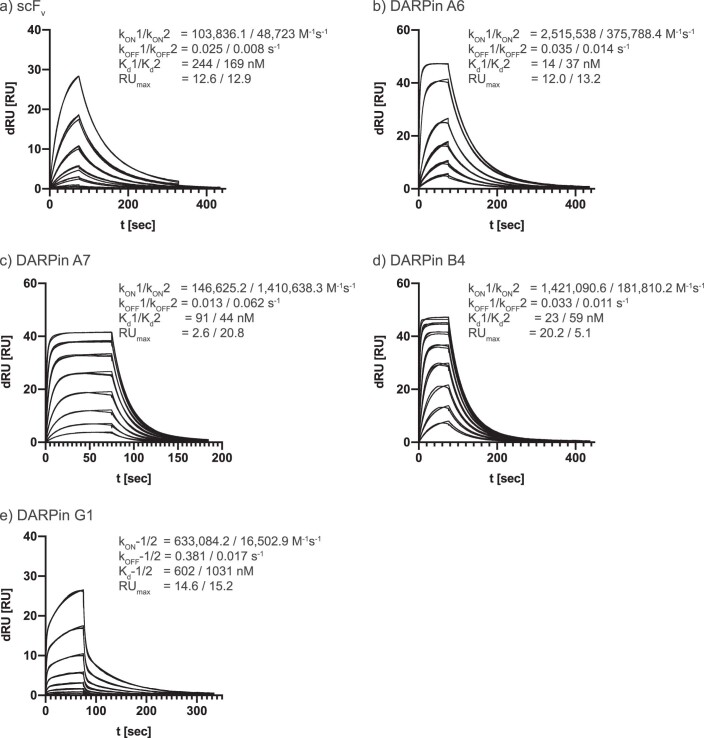
SPR measurements of scFv and DARPin binders. Affinities of binders towards the xFDP preparation used for DARPin selection were assessed by surface plasmon resonance (SPR) measurements on a Biacore T200 (GE Healthcare) using a CMD200M chip coated with 205 RU of xFDP protein. Due to the symmetric structure of the target molecules and thus a potential interaction between the binders on the ligand, we stipulated a heterogenous binding modality, resulting in two distinct binding constants. Affinities of G1-DARPin were up to 10- to 50-fold lower than those of other DARPins may explain the poor performance of G1-DARPin in a receptor context. Duplicates of full kinetic measurements were performed and fitted with a heterogenous ligand (hetlig) model using the BIAevaluation software (GE Healthcare). Flow cell 1 (FC1) served as a reference for FC2 and was coated with 205 RU of xFDP via NHS linkers. Each concentration was measured twice in ascending and descending order. Source Data

**Extended Data Fig. 5:**
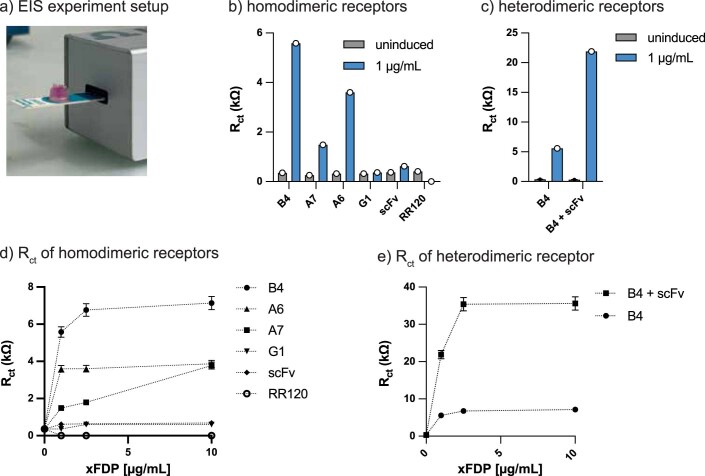
Verification of binding of xFDPs to receptors by electrochemical impedance spectroscopy (EIS). Binding of xFDPs to receptors was verified by electrochemical impedance spectroscopy (EIS). A three-electrode system was employed (Metrohm DS - 220AT) to sense changes in the charge transfer resistance (R_ct_) of the redox couple [Fe(CN)_6_]^3−^/[Fe(CN)_6_]^4−^ caused by binding of xFDPs to the receptors which insulates the electrode and hampers electron flow. Higher R_ct_ values indicate an increase in resistance and are indicative of higher affinity of the receptor, which translates to increased sensitivity. a) Cells transfected with receptors were incubated in the presence of [Fe(CN)_6_]^3−^/[Fe(CN)_6_]^4−^, and changes in R_ct_ were recorded upon addition of 1 µg/ml of xFDPs to b) homodimeric or c) heterodimeric receptors. b) Homodimeric receptors based on DARPins A6, A7, B4, G1 and the scFv showed R_ct_ values of 3.59 kΩ, 1.48 kΩ, 5.58 kΩ, 0.362 kΩ and 0.62 kΩ, respectively. c) The heterodimeric B4 + scFv receptor showed a strong activation (Rct = 21.88 kΩ). R_ct_ values for 1, 2.5 and 10 µg/ml of xFDPs measured for d) homodimeric receptors and e) B4 + scFv heterodimeric receptor seem to saturate at 2.5 µg/mL for all receptors, except for the homodimeric DARPin A7 receptor. Marginal increase in R_ct_ of RR120 control receptor can be attributed to non-specific binding. All values are means ± SD of n = 3 independent ssamples. Source Data

Binding affinities of purified binders for xFDPs were measured by means of surface plasmon resonance (SPR) assuming a heterogenous binding model, which yielded two distinct binding constants; these were 169/244 nM for the scFv and 14/37 nM, 44/91 nM, 23/59 nM, and 602/1030 nM for DARPins A6, A7, B4, and G1, respectively (Extended Data Fig. 4). We also confirmed that the binders are functional in a receptor context by employing electrochemical impedance spectroscopy (EIS) to measure changes in charge transfer resistance (R_ct_) upon ligand binding using an electrochemical sensor system^26^. For a fixed concentration of 1 µg ml^−1^ xFDPs, homodimeric receptors based on DARPins A6, A7, B4, G1, and the scFv showed R_ct_ values of 3.59 kΩ, 1.48 kΩ, 5.58 kΩ, 0.362 kΩ, and 0.62 kΩ, respectively. The heterodimeric B4 + scFv receptor showed strong activation (R_ct_ 21.88 kΩ) by 1 µg ml^−1^ xFDPs, mirroring the higher affinity quantified by SPR measurement (Extended Data Fig. 5 and Supplementary Fig. 8).

### Determining fibrinogen activation as root cause of induction

To characterize the impact of potentially underrepresented xFDP species in the initial DARPin selection as compared to the native plasma environment (Extended Data Fig. 1) and to exclude off-target receptor activation, we next investigated the receptor sensitivity to human plasma and tried to identify the active molecule(s) for AMBER_B4_, AMBER_B4/scFv_, AMBER_G1-B4_, and AMBER_scFv-B4_.

**Fig. 4:**
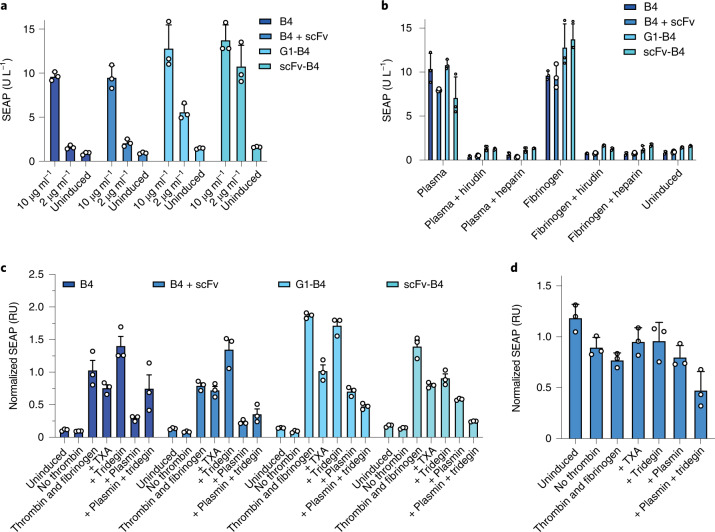
Characterization of specificities of selected AMBERs towards plasma. **a**, Reactivity of receptors towards fibrinogen was tested in a minimal system using two different concentrations of fibrinogen and thrombin. **b**, Reactivity towards fibrinogen and plasma can be blocked by addition of known anti-coagulants heparin and hirudin. **c**, Cross-linking of fibrinogen is mediated by factor XIII (FXIII) and blocked by its inhibitor tridegin. Reporter expression is modulated by addition of tridegin or plasmin and plasmin inhibitor TXA in a minimal system comprising purified fibrinogen and thrombin. Reporter activity is normalized to a sample with constitutive expression of the reporter under the same conditions to compensate for unspecific alterations. **d**, Values for B4 + scFv shown in **c** are normalized to values for B4 to emphasize differences in reporter expression between both receptor configurations. All values are mean ± s.d. of *n* = 3 independent samples. Source Data

To this end, we characterized the receptor responses to purified fibrinogen (Fig. 4a), plasma fractions obtained by SEC and analyzed by mass spectrometry (Supplementary Fig. 9), and purified fragments D and E of fibrinogen (Supplementary Fig. 10). We also investigated the effect of plasmin on fibrinogen (Supplementary Fig. 11a). We found that activation of receptors by fibrinogen or plasma requires the presence of FCS containing active thrombin (Fig. 4b and Supplementary Fig. 12a) to produce active molecules from matured fibrin (Supplementary Fig. 12b,c), that inducer molecules are final products (Supplementary Fig. 11b) of fibrin degradation, and that receptors are not activated by plasmin cleavage products of unprocessed fibrinogen (Supplementary Fig. 11c).

**Extended Data Fig. 6:**
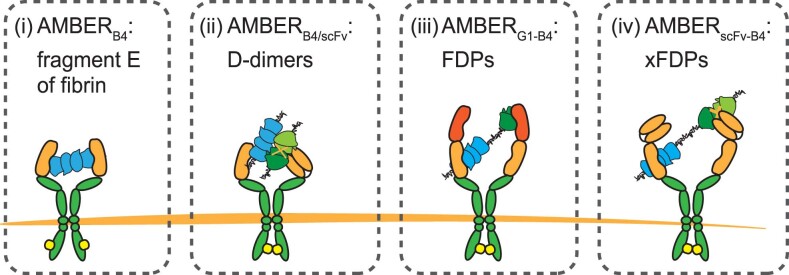
Summary of suggested receptor specificities. Summary of suggested receptor specificities. AMBER_B4_ may preferentially bind to fragment E of fibrin, AMBER_B4/scFv_ may bind to cross-linked D-dimers complexed with fragment E (DD-E complex) or fragment E alone (because some parts are also presented in the AMBER_B4_ configuration), AMBER_G1-B4_ may bind partially to non-cross-linked fibrin degradation products (FDPs), and AMBER_scFv-B4_ favors cross-linked FDPs (xFDPs).

We also investigated the effect of plasmin, its inhibitor tranexamic acid (TXA), and leech-derived peptide inhibitor of cross-linking factor XIIIa, tridegin, on coagulation-mediated receptor activation (Fig. 4c,d and Supplementary Fig. 13). In summary, we conclude that DARPin-B4 (present in AMBER_B4_ and AMBER_B4/scFv_) binds FDPs in a cross-linking-independent manner, and possible binding partners are fragment E as a single component or as a part of D–E or DD–E complexes (Extended Data Fig. 6). In line with the original publication^24^, we found that the scFv moiety within AMBER_B4/scFv_ binds cross-linked D-dimer protein in complex with fragment E (DD–E complex), which in turns binds to B4-DARPin (Extended Data Fig. 6). Receptors based on the scFv alone cannot detect D-dimers or coagulation (compare Fig. 3a,d). Nevertheless, we can build functional receptors that combine DARPin binders and the scFv.

The tandem receptor AMBER_scFv-B4_ detects larger partially digested and preferentially cross-linked FDPs while AMBER_G1-B4_ preferentially binds to non-cross-linked fragments (Fig. 4c,d and Extended Data Fig. 6). In conclusion, AMBERs in the analyzed set have different sensitivities and specificities towards fibrin degradation products, illustrating the versatility of the AMBER platform.

**Extended Data Fig. 7:**
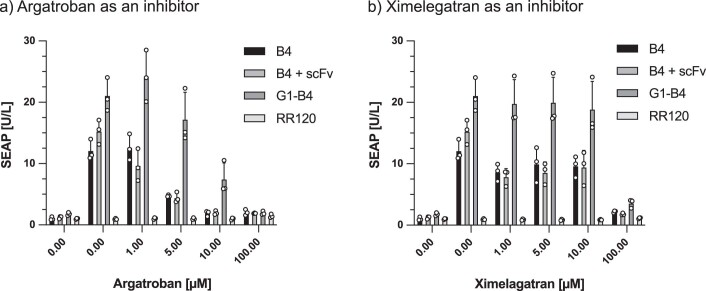
Benchmarking of established inhibitors of coagulation. Benchmarking of established inhibitors of coagulation. Cells were incubated for 24 h in complete DMEM containing plasma or fibrinogen and either 1 µg/mL hirudin or 15 USP/mL heparin. Inhibition of coagulation by the thrombin inhibitors b) argatroban and c) ximelegatran was assessed by measuring the SEAP reporter production of cells expressing AMBER_B4_, AMBER_B4/scFv_ or AMBER_G1-B4_. All values are means ± SD of n = 3 independent samples. Source Data

Even recently developed anti-coagulative drugs are constitutively active and hence increase the risk of bleeding, or can induce thrombocytopenia^27,28^. An integrated cellular system able to measure efficacy and toxicity in parallel, using the DARPins and receptors developed here, could shorten the development time of new anti-coagulant therapies. We confirmed that our system responds dose dependently to the thrombin inhibitor argatroban, in agreement with findings in a fibrin-bound thrombin system^29^ (Extended Data Fig. 7a), while the anti-coagulant prodrug ximelagatran remained ineffective at concentrations up to 100 µM (Extended Data Fig. 7b).

### Detection of pathological events in patients’ blood

**Fig. 5:**
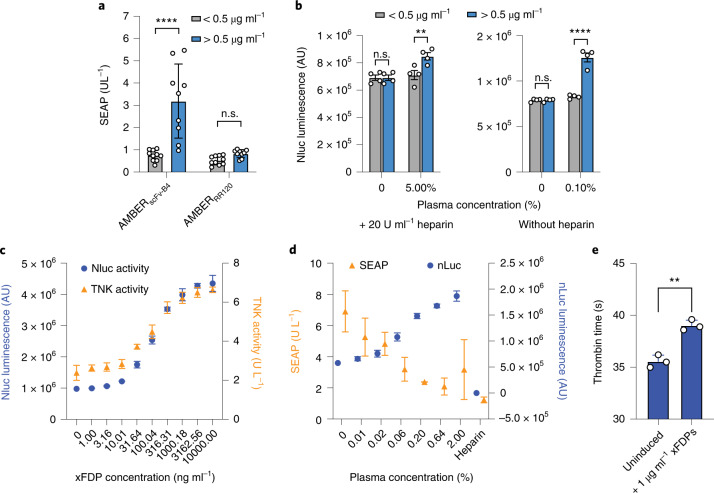
Evaluation of FDP-AMBERs using clinical samples and therapeutic outputs. **a**, AMBER_scFv-B4_ was induced with plasma samples of 19 donors (5% (v/v), supplemented with 20 U ml^−1^ of heparin) from patients with elevated D-dimer levels as measured by day-to-day clinical assessment (*n* = 9). The results were compared with control samples with normal levels (*n* = 10). The data suggest that the AMBER_scFv/B4_ can detect elevated levels of D-Dimers in patient samples (*P* < 0.0001). n.s., not significant. **b**, Discrimination of plasma samples (5% v/v plasma) of *n* = 4 patients with DIC with D-dimer levels >0.5 mg l^−1^ from *n* = 4 control samples (D-dimer < 0.5 mg l^−1^) by AMBER_scFv/B4_ either with heparin supplementation to assess the response to xFDPs (left) or without heparin (right) to test detection of the hypercoagulable state underlying DIC. The system was able to discriminate samples taken from diseased patients from those of donors showing normal D-dimer levels on the basis of D-Dimer levels (left; *P* = 0.0049) and hypercoagulability (right; *P* < 0.0001). **c**, Measurements of xFDP-induced expression of reporter Nluc and TNK from HEK_scFv-B4/TNK_ cells show the two are well correlated and legitimates the use of Nluc reporter production as a proxy for TNK activity. Forty-five thousand cells were seeded per well and incubated for 24 h. We subsequently induced those cells for 24 h with the indicated concentrations of xFDP protein or plasma. Nluc and TNK activity were measured in the supernatant. TNK activity of untreated cells was subtracted from the observed values. **d**, Hirudin-HM2-induced inhibition of coagulation is sensed by reporter cells equipped with AMBER_B4_ and expressing SEAP in response to coagulation (Extended Data Fig. 4). mHEK_B4/scFv/hirudin_ cells show a clear correlation of induction by higher concentrations of xFDP protein with reduced coagulation. **e**, The thrombin time of mHEK_B4/scFv/hirudin_ cells induced with xFDPs was assessed in a clinical laboratory and compared with that of the uninduced control (*P* = 0.0016). Values show mean ± s.d. of 10 (control) and 9 (elevated) patient samples (**a**), *n* = 4 patient samples for each condition (**b**), *n* = 4 independent samples (**c**). We performed statistical tests using a two-way analysis of variance with Šídák’s multiple comparisons test (**a**,**b**) and a two-tailed unpaired parametric *t*-test (**e**). Source Data

We next sought to validate our system using plasma from patients with elevated or physiological D-dimer concentrations. AMBER_scFv-B4_ could detect pathological D-dimer levels in patients’ samples supplemented with heparin (*P* < 0.0001 versus negative control AMBER_RR120_; Fig. 5a). Additionally, AMBER_B4/scFv_ could detect both elevated D-dimer levels (Fig. 5b; *P* = 0.0049) and a hypercoagulable state^30^ leading to depletion of anti-coagulative factors^31^ in patients with disseminated intravascular coagulation (DIC) (Fig. 5b; *P* < 0.0001 versus normal D-dimer levels in samples without heparin).

### Generating designer cells to counteract coagulation

To expand the functionality of the D-dimer-sensing receptors we constructed mammalian designer cells able to sense coagulation or elevated xFDPs and respond by secreting an anti-thrombotic protein, TNK or hirudin. We employed both the more sensitive heterodimeric AMBER_B4/scFv_ and the genetically more compact homodimeric AMBER_scFv-B4_ tandem receptor (compare Fig. 3b,c). Among reporters containing two, four, 12, or 16 repeats of the response element (Supplementary Fig. 14a), four repeats proved optimum.

**Extended Data Fig. 8:**
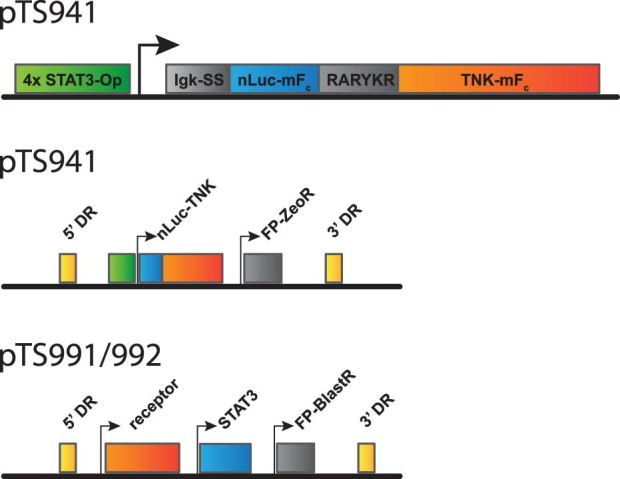
Schematic illustration of vectors used for the generation of stable coagulation-sensing cells. Schematic illustration of vectors used for the generation of stable coagulation-sensing cells. Top: sensing and expression cassette for a secreted nano-luciferase (Nluc) reporter fused to a murine F_c_ tag for enhanced stability in vivo (Nluc-mFc) and a tenecteplase-mFc fusion construct (TNK-mF_c_) used in pTS992 (RE_4_-P_hCMV_-IgkSS-Nluc-mF_c_-RARYKR-TNK-mF_c_-P_hCMV_-ZeoR-p2a-YPet-pA). Equimolar production and separation of the products during secretion are ensured by placing a furin cleavage site (RARYKR) between the two proteins. Middle: construct for stable integration of the reporter cassette using the Sleeping Beauty transposon system expressing a fluorescent protein (FP) to facilitate fluorescence-activated cell sorting (FACS) coupled to antibiotic resistance markers for zeocin (ZeoR) or blasticidin S (BlastR) for stable cell line selection. Bottom: schematic of constructs pTS941 (P_hCMV_-D-dimer-scFv-DARPinB4(tandem)-receptor-pA-P_SV40_-STAT3-pA-P_hCMV_-TagBFP2-p2a-PuroR-pA) and pTS942 (P_hCMV_-RR120-scFv-receptor-pA-P_SV40_-STAT3-pA-P_hCMV_-TagBFP2-p2a-PuroR-pA) for stable integration of the receptors as well as an overexpression module for signal transducer and activator of transcription 3 (STAT3) transcription factor. DR, direct repeat; Nluc, nano-luciferase; TNK, tenecteplase (htPA variant); mF_c_, murine antibody fragment for increased stability.

On the basis of this reporter, we generated a plasmid for genomic integration of a reporter cassette for induced expression of a secreted version of nano-luciferase (Nluc) fused to TNK via a furin cleavage site (RARYKR) (Extended Data Figure 8). The furin site is cleaved in the Golgi apparatus to deliver the two proteins to the supernatant in equimolar ratio so that reporter activity is correlated with potential therapeutic effect. Two stable monoclonal HEK-293T cell lines were selected and termed HEK_scFv-B4/_TNK (active receptor) and HEK_RR120R/TNK_ (negative control) (Supplementary Fig. 14b). HEK_scFv-B4/TNK_ cells showed a good correlation between TNK and Nluc in the supernatant of monoclonal HEK_scFv-B4/TNK_ in response to xFDPs (Fig. 5c). The onset time was 6 h, within the expected limits of a transcription-based gene switch (Supplementary Fig. 14c,d). Notably, the system detected xFDPs at levels as low as 0.1 µg ml^−1^, well below the clinical disease threshold (0.5 µg ml^−1^), and was sensitive to plasma similar to transient transfections (Supplementary Fig. 14e,f).

### Preventing coagulation with hirudin-producing designer cells

**Extended Data Fig. 9:**
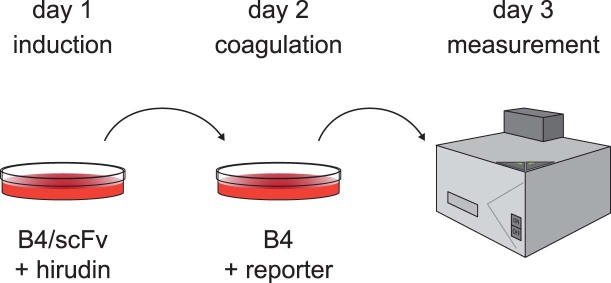
Workflow to assess hirudin-HM2 efficacy in cell culture. Workflow to assess hirudin-HM2 efficacy in cell culture. mHEK_B4/scFv/hirudin_ cells were induced with xFDP protein on day 1. Supernatant of induced mHEK_B4/scFv/hirudin_ cells was mixed with 0.5% (v/v, final) plasma on day 2 and dispensed on coagulation-sensing cells expressing AMBER_B4_. SEAP reporter expression from sensor cells and Nluc activity of mHEK_B4/scFv/hirudin_ are measured in the same sample with a multiplate reader on day 3.

Hirudin^32^ and heparin^33^ or its derivatives are often used as anti-coagulants. Hence, we replaced the reporter construct used for inducible tenecteplase expression with an Nluc-p2a-hirudin sequence (secreted hirudin-HM2 from *Poecilobdella manillensis)*. We generated a stable monoclonal HEK-293T cell line (mHEK_B4/scFv/hirudin_) harboring the new reporter construct alongside AMBER_B4/scFv_. To assess the efficacy of the produced hirudin, we employed the AMBER_B4_-based coagulation-reporter system. Monoclonal mHEK_B4/scFv/hirudin_ cells were induced with xFDPs to co-express hirudin and NLuc enabling quantification of bioluminescence as a proxy for hirudin (Extended Data Fig. 9). After 24 h, the hirudin- and NLuc-containing supernatants were transferred to AMBER_B4/SEAP_ cells. To profile the anti-coagulant effect of hirudin the reporter cell culture was supplemented with 0.5% (v/v) reconstituted human plasma. Purified hirudin and heparin were used as controls. Profiling hirudin production via bioluminescence and coagulation via AMBER_B4_-triggered SEAP expression enabled the quantification of the anti-coagulant impact of hirudin. Hirudin expression reduced the coagulation-induced signal of the reporter cells by 3.3-fold (Fig. 5d), and significantly (*P* = 0.0016) prolonged the thrombin clotting time by 10% (Fig. 5e), demonstrating functional secretion of the therapeutic protein.

### Detection of coagulation events in vivo

**Fig. 6:**
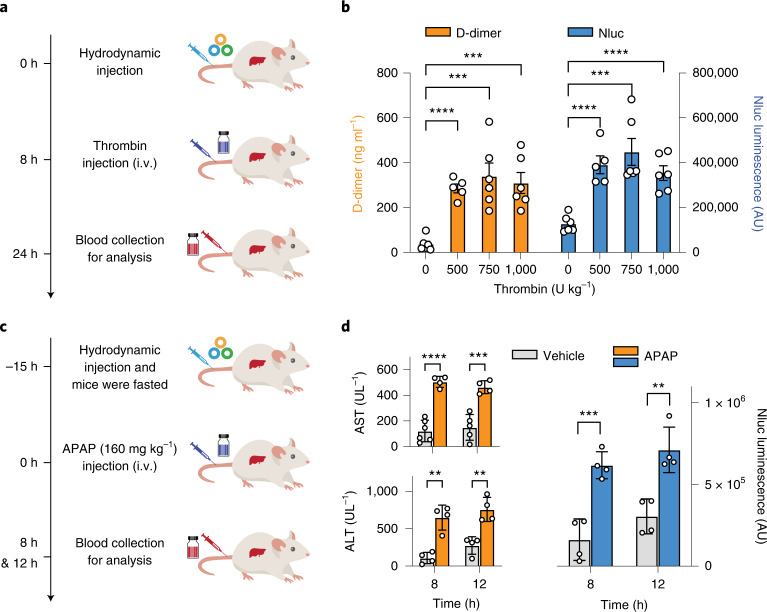
Confirmation of AMBER function in mouse models of systemic and local thrombotic events. **a**,**b**, Activation of transgene expression by coagulation-responsive AMBER_B4/scFv_ in mice. **a**, Schematic of the experimental design for transduction, induction of coagulation, and measurement of transgene expression in mouse liver. Coagulation-responsive AMBER_B4/scFv_, and a Nluc reporter plasmid were hydrodynamically injected via the tail vein. Eight hours later, mice were injected with different dosages of thrombin (0–1000 U kg^−1^) via the tail vein. **b**, Blood D-dimer and NanoLuc levels were quantified at 16 h after thrombin administration and found to be significantly increased in comparison to untreated controls (*P* values <0.0001, 0.0005, and 0.0002 (D-dimer); *P* values <0.0001, 0.0004 and <0.0001 (NanoLuc)). **c**,**d**, Activation of transgene expression by AMBER_B4/scFv_ upon liver damage. **c**, Experimental setup of the mouse model of acute hepatic injury. Wild-type male 8-week-old C57BL/J6 mice were hydrodynamically transduced with AMBER_B4/scFv_. Fifteen hours after plasmid injection, fasted mice were intraperitoneally injected with APAP (0 or 160 mg kg^−1^). **d**, AST and ALT levels were measured to confirm the effect of APAP treatment and NLuc activity indicates reporter cell induction. Blood AST, ALT, and NLuc levels were significantly increased at 8 h and 12 h post intraperitoneal APAP injection (AST 8h *P* = 0.000064; AST 12h *P* = 0.000736; ALT 8h *P* = 0.002039; ALT 12h *P* = 0.002999; NLuc 8h *P* = 0.001878; NLuc 12h *P* = 0.003533). Data are expressed as mean ± s.e.m. of five or six mice. Two-tailed unpaired parametric *t*-tests were used for statistical analysis. Source Data

In a proof-of-concept study, we assessed whether AMBER_B4/scFv_ can be dose dependently activated in mouse models of induced systemic coagulation and acute hepatic injury. Homodimeric AMBER_A7_ and AMBER_B4_ are activated by both human and mouse plasma in vitro, while AMBER_A6_ was activated only by human plasma (Supplementary Fig. 15). Hence, in all mouse experiments we used the AMBER_B4/scFv_ receptor alongside a STAT3-responsive NLuc reporter. The system was hydrodynamically injected via the tail vein to transduce the liver, and coagulation was induced 8 h later by injection of 500, 750, or 1000 U kg^−1^ thrombin (Fig. 6a). After 24 h, expression of the reporter transgene correlated with D-dimer levels, indicating successful implementation of the AMBER_B4/scFv_ system in vivo (Fig. 6b). Next, we evaluated the system in a mouse model of acute hepatic injury^34^ caused by acetaminophen (N-acetyl-*p*-aminophenol (APAP)), a leading cause of acute liver damage in humans^35^. APAP-induced liver injury leads to activation of coagulation and hepatic fibrin deposition within 2–6 h in mice. Measurements of blood levels of aspartate aminotransferase (AST) and alanine aminotransferase (ALT) at 8 h and 12 h after intraperitoneal APAP injection confirmed liver damage (Fig. 6c), and elevated levels of Nluc demonstrated that the AMBER sensor was activated (Fig. 6d).

## Discussion

In this work we have combined DARPin selection with our GEMS technology, and we show that the resulting AMBER platform enables the generation of programmable receptors with customizable sensitivity and specificity by screening a vast space of possible epitopes. Such versatility is unprecedented in a receptor system for soluble ligands. We confirmed that this modular receptor system works as expected for FDPs, which we chose as markers with high clinical relevance for proof of concept. Coagulation was detected at an early stage before the build-up of insoluble fibrin by employing the AMBER_B4_ system, and we could detect early as well as late biomarkers of thrombotic events by using an integrated sensor incorporating a previously characterized scFv (AMBER_B4/scFv_ or AMBER_scFv-B4_). Furthermore, AMBER receptors were coupled to therapeutic protein output, either tenecteplase to boost clot lysis or hirudin to prevent further coagulation, in designer cell lines. Secretion of a therapeutic protein alongside a bioluminescent reporter provided a direct correlation of reporter activity with expression of the therapeutic protein. Finally, we established that the system is applicable for the detection of thrombotic events in patients, as well as in mouse models in which coagulation is induced either systemically, mimicking DIC, or triggered locally by APAP-induced liver injury. These results suggest that the AMBER pipeline has translational potential for cell-therapy approaches to treat recurrent thrombosis or embolism.

In addition, the selected anti-FDP DARPins may be useful to build new diagnostic tools or therapeutic agents, by expanding the available epitope landscape for the development of ELISA-like assays or DARPin-coupled fibrin-targeted drugs for clot lysis. Owing to its shorter half-life, fragment E (as presumably targeted by some of the DARPins in this study) might provide a more precise insight into the progression of thrombotic diseases as compared to other degradation products such as D-dimers^36,37^.

Notably, not all hits from the initial DARPin selection turned out to be good candidates for building a functional receptor, highlighting the importance of a diverse library as input for the AMBER pipeline—especially since sandwich pairs, both having sufficient affinity, and a suitable relative disposition of epitopes are required. Indeed, for induction by plasma, about 16% of combinations with a known scFv and 40% of DARPin-only receptors were active in a heterodimeric receptor configuration. Since about 83% of these DARPin-only receptors are based on one of four of the initially characterized 19 DARPins, the overall success rate in finding suitable binders was 21%. With a symmetric target, the generation of homodimeric receptors is much more likely, and we thus expect the likelihood of finding such receptors for monomeric targets to be lower. Nevertheless, the GEMS platform has been successfully used in combination with pre-existing binders to detect the monomeric 26 kDa cancer antigen PSA with the use of two scFvs^15^.

Confirmation of the binding of xFDPs to homodimeric receptors by EIS means that the OFF-type switching observed in the reporter gene assays (Fig. 3a) can indeed be attributed to inactivation of homodimeric receptors by ligand binding. Also, binding of xFDPs is much stronger in a heterodimeric setting (B4 + scFv) than in the homodimeric setup (B4 or scFv alone), indicating a non-linear effect of receptor–ligand interaction, possibly owing to increased avidity as suggested by SPR quantifications (Extended Data Figs 8 and 9).

As elaborated on earlier, we expect the number of working combinations of FDP binders that satisfy the design constraints of the receptor architecture to be low owing to size restrictions. From a reported crystal structure of activated EpoR (PDB ID: 1EER), as well as the results of the GFP-AMBER experiments we can estimate the distance between two epitopes to range from ~8 nm (distance between the ends of the D1 domain of activated EpoR comprising the linker length and the size of GFP) to ~3 nm (two times half the length of the GFP barrel (PDB ID: 5B61) in the GFP–GFP fusion without any linker). Of course, the geometry of the receptor–ligand interaction should be taken into account^18^, but different binder types would allow for different geometries.

While one can build receptors using pre-existing binders, an integrated pipeline to check a receptor library for activity in cell culture greatly extends the options, is easy to parallelize and is time- and cost-efficient. Therefore, we expect the versatile and highly streamlined DARPin technology in combination with the AMBER technology to provide access to large numbers of receptors for many targets, including soluble cancer antigens, cytokines or even cell-surface epitopes. AMBER might be especially useful for targets for which no natural receptor is known and for which available sequences of scFvs or other binders are limited, especially since sandwich pairs are needed. Modular switching of the signaling domains of the underlying receptor scaffold has already been reported^15^ and could be used to accelerate research in systems and stem cell biology to rewire endogenous signaling cascades.

The ribosome display technology coupled with the DARPin libraries used in this work provides a powerful method to generate highly diverse sets of binders through enrichment of high-affinity binders in a cell-free setup, and expanding the receptor platform to include other types of binders with different epitope preferences further broadens the range of possible epitopes.

While the DARPins generated in our screen have a concave interaction surface, other scaffolds mainly interact via protruding loops. Hence, we believe that different binder types can complement each other in sandwich pairs to facilitate the generation of AMBERs. The benefits of combining different binder types are already apparent in the present study in terms of the clear change in specificity observed upon employing an scFv in combination with a DARPin.

## Methods

### Chemicals and proteins

Native human D-dimer protein (ab98311), recombinant hirudin protein (ab201396) and native human plasmin (ab90928) were purchased from Abcam. *Trans*-4-(Aminomethyl)cyclohexanecarboxylic acid (tranexamic acid, TXA, 857653), heparin sodium salt from porcine intestine (H3149), lyophilized human plasma (P9523), active thrombin from human plasma (SRP6557), and fibrin from human plasma (F5386) were purchased from Sigma-Aldrich. Human fibrinogen fragment E (HCI-0150E) and human fibrinogen fragment D (HCI-0150D) were purchased from Haematologic Technologies. Soluble human fibrin (FIB-S-1.0MG) was purchased from Molecular Innovations Tridegin (opr0062) was purchased from Covalab S.A.S.

### Selection and screening of DARPin binders specific for FDP

To generate DARPin binders for human FDP protein, FDPs were biotinylated using EZ-Link NHS-Biotin (ThermoFisher Scientific) and the biotinylated target protein was immobilized on either MyOne T1 streptavidin-coated beads (Pierce) or Sera-Mag neutravidin-coated beads (GE), depending on the selection round. Ribosome display selections were performed essentially as described^11^, using a semi-automatic KingFisher Flex MTP96 well platform.

For the present work, we used an established, optimized, fully synthetic DARPin library in which the backbone of the DARPin scaffold remained constant and the amino acids forming the target-interacting surface were randomized. The design strategy and characteristics of this library, which has a theoretical diversity of 3.8 × 10^23^ unique sequences, have been reviewed elsewhere^13^. Because of the high diversity of the library and the limited number of actual molecules present in each ribosome display run (estimated to be ‘only’ 10^12^), essentially no duplicates are screened, as verified by sequencing, and this also allows for repeated screens against the same target to yield different binders.

We started screening with a synthetic library comprised of DARPins with three internal repeat domains and distinct N-terminal and C-terminal capping repeats (N3C-DARPins), where the terminal repeats were designed with or without randomization of the predicted binding interface^13^. This plasmid DARPin library was transcribed into mRNA, and the obtained mRNA was translated using a cell-free extract^11^. Since the mRNA lacks a stop codon, translation is stalled at the last codon, because the ribosome can neither progress nor can the polypeptide be cleaved from the tRNA. Consequently, the mRNA is attached through the ribosome to the corresponding nascent peptide. Because of extra residues at the C terminus (the ‘tether’) this polypeptide folds into a functional DARPin that can bind to immobilized xFDPs coupled to streptavidin-coated beads through biotinylated lysine residues. The resulting xFDP-DARPin-ribosome-mRNA complexes are enriched using magnetic beads and disassembled to extract the enriched mRNA. The selected mRNA is used in a subsequent cycle to further increase the affinities of DARPins in the pool. This sequence of reactions allows for the selection of the highest affinity binders from the initial library, as described below.

The fully synthetic library consists of N3C-DARPins with three randomized internal repeats, containing a mixture of non-randomized and randomized N-terminal and C-terminal capping repeats^10,13,38^. Selections were performed over four rounds with decreasing concentrations of xFDPs for the first three cycles, an off-rate selection using non-biotinylated xFDPs in the third cycle and a recovery phase with less stringent conditions in the fourth cycle^12,39^.

The final enriched pool was cloned as fusions into a bacterial pQE30 derivative vector with a N-terminal MRGSH_8_ and C-terminal FLAG tag via unique BamHI and HindIII sites, containing lacIq for expression control. After transformation of *E*. *coli* XL1-blue, 380 single DARPin clones were expressed in 96-well format and lysed by addition of a concentrated Tris-HCl-based HT-Lysis buffer containing *n*-octyl β-d-thioglucopyranoside, lysozyme and universal nuclease (Pierce). These bacterial crude extracts of single DARPin clones were subjected to HTRF-based screening to identify potential binders. For this purpose, binding of the FLAG-tagged DARPins to streptavidin-immobilized biotinylated FDP protein was measured using FRET (donor: streptavidin-Tb, acceptor: anti-FLAG-d2; Cisbio). Further HTRF cross-reactivity measurements against ‘No Target’ and HSA allowed for discrimination of FDP-specific hits.

Among the identified binders, 32 were sequenced and 19 single clones were selected. The DARPins were expressed on a small scale and purified using a 96-well IMAC column (HisPur Cobalt plates, Thermo Scientific). DARPins after IMAC purification were analyzed at a concentration of 10 µM on a Superdex 75 5/150 GL column (GE Healthcare) using an Äkta Micro system (GE Healthcare) with PBS containing 400 nM NaCl as the running buffer. Absorbance at 280 nm was recorded. β-amylase (200 kDa), bovine serum albumin (66 kDa), carbonic anhydrase (29 kDa) and cytochrome c (12.4 kDa) were used as molecular mass standards. 15 DARPins were identified as monomers.

### Sequence analysis

Phylogenetic analysis of amino acid sequences was done with a simple phylogeny tool (https://www.ebi.ac.uk/Tools/phylogeny/simple_phylogeny/). Sequence alignments were performed using Clustal O (https://www.ebi.ac.uk/Tools/msa/clustalo/). Pairwise identity calculation was carried out using the EMBL-Needle algorithm employing the BLOSUM62 matrix (https://www.ebi.ac.uk/Tools/psa/emboss_needle/). All algorithms are part of the EMBL-EBI search and sequence analysis tools API^40^.

### Plasmid preparation

A comprehensive list of all plasmids used in this study is provided in Supplementary Table 1.

Plasmids were generated using conventional molecular cloning techniques. Polymerase chain reaction (PCR) was performed using Phusion polymerase (F530, ThermoFisher Scientific) or Q5 high-fidelity polymerase (M0491, New England Biolabs, NEB) following the manufacturers’ recommendations. Cleavage of PCR products and plasmids was done with restriction endonucleases (New England Biolabs NEB, HF enzymes were used if applicable) and ligated with T4 DNA ligase (EL0011, ThermoFisher Scientific).

### SDS-PAGE and Coomassie staining

Samples were heated for 15 min at 70 °C before loading. Up to 20 µl sample was loaded per well of a 12-well or 15-well polyacrylamide gel (Bolt 4–12% Bis-Tris Plus gels, Invitrogen). SDS-PAGE was performed by using 1× Bolt MOPS SDS Running Buffer (Invitrogen) in a XCell SureLock Mini-Cell (Invitrogen) following the instructions of the manufacturer. PageRuler Plus Prestained Protein Ladder (Invitrogen) was used as a size reference.

Gels were washed three times with water after the run to remove SDS from the running buffer, then stained with Coomassie (17% (v/v) methanol, 3.3% (v/v) acetic acid, and 0.08% (w/v) Coomassie Brilliant Blue G250) for >1 h at room temperature. Gels were washed and destained in water for several days before mass-spectrometric analysis.

### Cell culture

HEK-293T/17 (HEK-293T) cells (ATCC CRL-11268) were purchased from the American Type Culture Collection and grown in high-glucose Dulbecco’s modified Eagle’s medium containing GlutaMAX (DMEM, high glucose, GlutaMAX Supp, 61965026, ThermoFisher Scientific) supplemented with 10% fetal bovine serum (FBS, F7524, Sigma-Aldrich) at 37 °C in a humidified incubator with a 7.5% CO_2_ atmosphere. Cells were grown in 10 ml DMEM at a seeding density of 1.5 × 10^6^ cells in a 10-cm culture dish and kept at <80% confluency.

For transfection, cells were seeded in 15 ml DMEM supplemented with 1× penicillin/streptomycin (L0022, Biowest) in 96-well culture plates (15,000 cells per well). Stable HEK-293T cells were generated using the Sleeping Beauty transposon system^41^. Donor plasmids bearing a selection marker (resistance genes for puromycin (*pac*), blasticidin (*bsr*) or zeocin (*ble*)) driven by a constitutive promoter and flanked by recognition sites of the Sleeping Beauty transposase were co-transfected with an expression plasmid for Sleeping Beauty transposase. For selection of stable clones, the cell culture medium was supplemented with puromycin (2 µg ml^−1^), blasticidin (4 µg ml^−1^) or zeocin (20 µg ml^−1^) or combinations of them, depending on the types of plasmids to be integrated.

### Transfections and stable cell line generation

HEK-293T cells were seeded from the same batch on the evening before transfection in the same plate format. For most reporter assays, transfection was done in a 96-well plate by mixing 200 ng plasmid DNA and 1.2 µl of 1 mg ml^−1^ polyethyleneimine (PEI, 24765-1, Polysciences) per well in FCS-free DMEM if not indicated otherwise. The mixture was incubated for 5–10 min, added to each well and incubated with the cells overnight. For all transient expression experiments, 2 ng each receptor plasmid was mixed with 30 ng plasmid pLS13 and 30 ng pLS15. Amounts of DNA were adjusted to 200 ng using the inert filler plasmid pDF101. A comprehensive list of plasmids used in each experiment is provided in Supplementary Table 2.

For stable cell line generation, 6-well plates were used. Here 250,000 cells per well were seeded and transfected 24 h later. A total of 1 µg plasmid DNA was mixed with 6 µl 1 mg ml^−1^ PEI in FCS-free DMEM. For two vectors to be integrated, 300 ng each integration vector were mixed with 200 ng Sleeping Beauty expression vector pTS395 and 200 ng pDF101.

For three integration vectors, 200 ng each integration vector was mixed with 200 ng Sleeping Beauty expression vector pTS395 and 200 ng pDF101.

Cells were grown for 1 day after transfection before starting selection, depending on the selection marker used, with a final concentration of 2 µg ml^−1^ of puromycin, 4 µg ml^−1^ of blasticidin S or 20 µg ml^−1^ of zeocin.

Stable transgenic monoclonal cell lines were produced from mixed populations by FACS-mediated single-cell seeding using a BD Aria III FACS.

### Reporter assays

To measure the activity of SEAP, the increase of absorbance at 405 nm owing to hydrolysis of *para*-nitrophenyl phosphate (pNPP) in the cell culture supernatant was followed (1 reading per minute for 25 min). To this end, 60–100 µl supernatant was collected from each well and heat-inactivated for 30 min at 65 °C. One hundred eighty microliters of assay reagent containing 100 µl 2× SEAP buffer (20 mM homoarginine, 1 mM MgCl_2_, 21% (v/v) diethanolamine, pH 9.8), 20 µl pNPP substrate solution (20 mM, in 2× SEAP buffer) and 60 µl water were added to 20 µl heat-inactivated supernatant. The absorbance at 405 nm at 37 °C was monitored with a Tecan M1000 multiplate reader (Tecan AG) and used to calculate SEAP activity.

Cell culture supernatant was also used to determine the activity of secreted nano-luciferase with the Nano-Glo Luciferase Assay System (N1110, Promega). To this end, 7.5 µl cell culture supernatant was mixed with 7.5 µl buffer/substrate mix per well in a 384-well black-well plate. Luminescence of the reporter was measured using a Tecan M1000 multiplate reader (Tecan AG).

### GFP fusion protein production and purification

BL21-Gold (DE3) cells were transfected with plasmids containing the expression units controlled by a *lac* operator. After induction with 0.4 M isopropyl β-d-1-thiogalactopyranoside, expression was conducted for 16 h at 18 °C. Cells were harvested by centrifugation at 4,000*g* at 4 °C. Cell pellets were then resuspended in 50 mM Tris-HCl (pH 8.0), 400 mM NaCl (TBS400), supplemented with 3 mg ml^−1^ lysozyme (Sigma-Aldrich) and 100 µg ml^−1^ DNase I (Roche), and lysed by sonication. The lysates were centrifuged (21,000*g*, 30 min, 4 °C) and the supernatants were applied to Ni-NTA Superflow (Qiagen) metal ion affinity columns (4 ml). Each column was washed with 15 column volumes of 50 mM Tris-HCl (pH 8.0), 20 mM imidazole supplemented with 400 mM NaCl, 1 M NaCl or 20 mM NaCl. The proteins were eluted in 5 column volumes PBS (pH 7.4), 500 mM imidazole. The eluted proteins were transferred into dialysis tubes with a molecular weight cutoff of 6,000–8,000 Da, supplemented with 100 µg ml^−1^ TEV protease (GenScript), and dialyzed for 36 h at 4 °C in phosphate-buffered saline. TEV protease-cleaved His-tags and non-cleaved proteins were removed by applying the dialyzed samples to Ni-NTA Superflow metal ion affinity columns (4 ml). The flow-through fraction was collected and concentrated by ultrafiltration (Amicon Centrifugal Filter Units, Millipore). The concentrated protein was then run through a Superdex 75 10/300 GL column to remove oligomers and impurities. Protein concentrations were determined by UV-Vis spectroscopy and purity was confirmed by SDS-PAGE analysis.

### Size-exclusion chromatography of reconstituted plasma

Reconstituted human plasma was filtered through a 0.2-µm syringe filter to clear liposomes and loaded on a self-packed Sephadex-200 column equilibrated with D-PBS containing 2 mM EDTA to prevent clotting in the column. The run was performed in the same buffer and a total of 133 fractions of 1 ml were collected. Selected fractions were further tested for their activity in cell culture as well as analyzed with SDS-PAGE and western blotting.

### Mass spectrometry

In-gel digestion of relevant SDS-PAGE bands was performed by using trypsin in 100 mM ammonium bicarbonate buffer. Samples were purified by using C18 columns, dried, and resuspended in 20 µl of a suitable buffer. Concentrations were adjusted to be approximately 0.1 µg µl^−1^ and 1 µg of the solution was subjected to mass-spectrometric analysis on an Orbitrap Elite system.

### Surface plasmon resonance measurement

Affinities of soluble binders were assessed using SPR using a CMD200M chip on a Biacore T200 (GE Healthcare) device. A reference flow cell (FC1) on the same chip was used as a control for the measurement flow cell (FC2). Both flow cells were coated with 205 RU of (x)FDP (D-dimer preparation) using NHS chemistry. Two runs of full kinetic measurements were performed, and results were fitted with a heterogenous ligand (hetlig) model with the BIAevaluation software (GE healthcare). We measured kinetics at each concentration two times in ascending and descending order.

### Electrochemical impedance spectroscopy-based sensor assay

HEK-293T cells were transfected overnight as described above with receptor chains for homodimeric or heterodimeric receptor systems. Screen-printed Au electrodes (DS 220 AT, Metrohm) organized in the form of a three-electrode system were deployed to carry out the sensing measurements using a CH Instruments Electro-chemical Workstation (USA). The surface of the working electrode was modified with 0.001 mg ml^−1^ multiwall carbon nanotubes (Sigma) and poly-l-lysine (Sigma). A reservoir (200 µl in volume) was designed around the working electrodes in order to facilitate cell seeding and limit cell migration to the counter or reference electrodes. Transfected cells were seeded at a concentration of 8,000 cells per milliliter in standard culture medium. Cells were incubated on the electrodes at 37 °C overnight to ensure proper adherence. During the experiment, the cell-covered electrodes were exposed to xFDPs at concentrations of 1, 2.5, or 10 µg ml^−1^. Measurements were carried out in the presence of 5 mM solution of the redox couple Fe(CN)_6_]^3−^/[Fe(CN)_6_]^4−^ (Sigma). The measurements were performed in EIS mode, wherein a potential of +0.2 V was maintained between the working and reference electrodes (Ag/AgCl). The acquired Nyquist plots for homodimeric and heterodimeric interactions were fitted using two different modified Randle’s circuits each for a particular system as depicted in Supplementary Fig. 8d,e to obtain the charge transfer resistance (R_ct_).

### tPA activity measurements

A commercial kit was used to assess tPA activity following the manufacturer’s guidelines (Biovision, #K178–100) with an adaptation of the protocol for 384-well plates. We adjusted the amount of sample to 6 µl and scaled down the amount of inhibitor cocktail and substrate mix accordingly to 2 µl each. Plates were centrifuged between steps at 250 r.c.f. to collect samples at the bottom of the wells. We followed the protocol to determine the activity of TNK by recording the absorbance of the emerging pNA product at 405 nm over 1 h. A calibration curve was prepared using authentic pNA.

### Measurement of thrombin clotting times

To measure the effectiveness of the anti-coagulant hirudin, we measured thrombin clotting times at the laboratory of the University Hospital Basel using an established Thrombin Time HemosIL assay (Werfen GmbH). To this end, 300 µl sample was mixed with 150 µl reference plasma (Werfen GmbH) and the onset of coagulation was determined on an ACL top 750 (Werfen GmbH) device.

### Induction using human plasma samples

Human citrate-treated plasma samples were collected independently at the Yangpu Hospital, China and University Hospital Basel, Switzerland. D-dimer levels of all samples were assessed in the hospitals. All work was approved by the Medical Ethics Committee of Yangpu Hospital affiliated with Tongji University (request ID: LL-2018-SCI-003) for the Yangpu group and the Ethikkommission Nordwest- und Zentralschweiz (EKNZ) (request ID: 2021-01998) for the Basel group. Cells shown in Fig. 4a were seeded and transfected as described above. In Fig. 4b, 50,000 HEK-293T cells were seeded per well of a 24-well plate, cultured overnight for 20 h, and transfected with 8 ng pTS864 (P_hCMV_-DARPinB4-EpoR-pA), 8 ng pTS922 (P_hCMV_-scFv-EpoR-pA), 120 ng pLS15 (P_hCMV_-STAT3-pA) and 120 ng pTS2245 (P_OSTAT3_-NLuc-pA). After 6 h, cells were trypsinized and reseeded in 96-well plates at 10,000 cells per well and cultured for 20 h. After this time, plasma samples of disease and control patients was added directly to the cell culture medium (either without supplements or with additional heparin (BBI, A603251-0001), final concentration: 20 U ml^−1^) at the indicated concentrations. The expression of NLuc reporter in the supernatant was measured after 18 h.

### Mouse experiments

Wild-type male 6-week-old BABL/c mice obtained from East China Normal University (ECNU) Laboratory Animal Center were used and kept in a pathogen-free environment. Mice were kept in a 12 h light/12 h dark cycle at room temperatures ranging between 18 °C and 23 °C and humidities between 40% and 60%.

### Thrombin-mediated transgene expression in mice

Two milliliters (10% of the body weight in grams) Ringer’s solution (147 mM NaCl, 4 mM KCl, 1.13 mM CaCl_2_) containing approximately 360 μg plasmids encoding the thrombosis sensor (pTS864 (P_hCMV_-DARPinB4-EpoR-pA), 30 μg; pTS922 (P_hCMV_-scFv-EpoR-pA), 30 μg; pJH6 (P_OSTAT3_-NLuc-pA::P_hCMV_-STAT3-pA), 300 μg) was hydrodynamically injected into each mouse via the tail vein within 3–5 s. After injection, mice were randomly divided into four groups. Eight hours after plasmid injection, mice were injected with thrombin from bovine plasma (Beyotime, cat. no. 2594047) at doses ranging from 0 to 1,000 U kg^−1^ via the tail vein to induce thrombus formation. At 16 h after thrombin injection, plasma samples were obtained by centrifugation (2,700*g* for 10 min) of clotted blood in blood collection tubes (BD, cat. no. 365974) for analytical assays. D-dimer levels were measured using a Mouse D-Dimer ELISA Kit (Elabscience, cat. no. E-EL-M0400) following the manufacturer’s instructions, and plasma NanoLuc levels were measured using a Nano-Glo Luciferase Assay kit (Promega, cat. no. N1120) according to the manufacturer’s instructions.

### AMBER activation in an APAP-induced acute hepatic injury mouse model

Two milliliters (10% of the body weight in grams) of Ringer’s solution (147 mM NaCl, 4 mM KCl, 1.13 mM CaCl_2_) containing 30 µg pTS864 (PhCMV-DARPinB4-EpoR-pA), 30 μg pTS922 (PhCMV-scFv-EpoR-pA) and 300 μg pJH6 (P_STAT3_-NanoLuc-pA::PhCMV-STAT3-pA) was hydrodynamically injected into mice via a tail vein within 3–5 s. After the injection, mice were immediately fasted and randomly divided into two groups. Fifteen hours after plasmid injection, fasted mice were intraperitoneally injected with APAP (0 or 160 mg kg^−1^, Absin, cat. no. abs815915–500mg). Blood AST, ALT and Nluc levels were quantified at 8 h and 12 h post intraperitoneal APAP injection. Eight and twelve hours after APAP injection, blood samples were taken, allowed to clot, and centrifuged (2,700*g* for 10 min) in blood collection tubes (BD, cat. no. 365974) for analytical assays. Plasma AST and ALT levels were measured using an AST detection kit (KHB; SHFDA: 20142400181) and an ALT detection kit (KHB; SHFDA: 20172400610) according to the manufacturer’s instruction. Plasma Nluc levels were measured using a Nano-Glo Luciferase Assay kit (Promega, cat. no. N1120) according to the manufacturer’s instruction.

### Statistics

Calculations were performed in Microsoft Excel for Mac 16.50. All statistical analyses were done with GraphPad Prism 9.1. Representative graphs showing *n* = 3 biologically independent samples are presented as bar diagrams ± s.d. Unless indicated otherwise, no statistical analysis was performed. Plots in Fig. 3 show the mean ± s.e.m. of *n* = 3 independent experiments performed in triplicate. To determine the level of significance in Fig. 5a,b, a two-way analysis of variance was performed with Šídák’s multiple comparisons test. For the animal experiments in Fig. 6b,d, each treatment group consisted of randomly selected mice (*n* = 5–6). The results are expressed as means ± s.d. The statistical parameters were calculated to be as follows. For Fig. 5a, AMBER_scFv-B4_, adjusted *P* < 0.0001, *t* = 6.396, degrees of freedom = 34.00; AMBER_RR120_, adjusted *P* not significant, *t* = 0.7959, degrees of freedom = 34.00. For Fig. 5b, left, 0%, adjusted *P* > 0.9999, *t* = 0, degrees of freedom = 12; 5%, adjusted *P* = 0.0049, *t* = 3.821, degrees of freedom = 12. For Fig. 5b, right, 0%, adjusted *P* > 0.9999, *t* = 0, degrees of freedom = 12; 0.1%, adjusted *P* < 0.0001, *t* = 0, degrees of freedom = 12.

To determine the level of significance in Fig. 5e and Fig. 6b,d two-tailed unpaired parametric *t*-tests were performed. For the animal experiments in Fig. 6b,d, each treatment group consisted of randomly selected mice (*n* = 5–6). The results are expressed as means ± s.e.m. The parameters for *t*-tests were calculated to be for Fig. 4f, *P* = 0.0016, *t* = 7.585, degrees of freedom = 4. For Fig. 6b (NLuc reporter), 500 U kg^−1^ versus 0 U kg^−1^, *P* < 0.0001, *t* = 6.597, degrees of freedom = 9; 750 U kg^−1^ versus 0 U kg^−1^, *P* = 0.0004, *t* = 5.194, degrees of freedom = 10; 1,000 U kg^−1^ versus 0 U kg^−1^, *P* < 0.0001, *t* = 6.224, degrees of freedom = 10. For Fig. 6b (D-dimer levels), 500 U kg^−1^ versus 0 U kg^−1^, *P* < 0.0001, *t* = 10.85, degrees of freedom = 9; 750 U kg^−1^ versus 0 U kg^−1^, *P* = 0.0005, *t* = 5.055, degrees of freedom = 10; 1,000 U kg^−1^ versus 0 U kg−1, *P* = 0.0002, *t* = 5.625, degrees of freedom = 10. Fig. 6d for ALT, 8 h, adjusted *P* = 0.002039, *t* = 5.936, degrees of freedom = 6; 12 h, adjusted *P* = 0.002999, *t* = 4.8, degrees of freedom = 6; for AST, 8 h, adjusted *P* = 0.000064, *t* = 8.363, degrees of freedom = 8; 12 h, adjusted *P* = 0.000736, *t* = 5.699, degrees of freedom = 7; for NLuc reporter, 8 h, adjusted *P* = 0.001878, *t* = 6.03, degrees of freedom = 6; 12 h, adjusted *P* = 0.003533, *t* = 4.642, degrees of freedom = 6.

### Ethics

Animal experiments were approved by East China Normal University (ECNU) Animal Care and Use Committee and were conducted in accordance with the Ministry of Science and Technology of the People’s Republic of China guidelines. The protocol (ID: m20191212) was approved by ECNU Animal Care and Use Committee. All mice were killed after completion of the experiments.

Experiments involving patients’ samples were approved by the Medical Ethics Committee of Yangpu Hospital affiliated with Tongji University (request ID: LL-2018-SCI-003) and the Ethikkommission Nordwest und Zentralschweiz (EKNZ) (request ID: 2021-01998). All human participants agreed to the use of their blood samples for research purposes and expressed their consent in writing.

### Reporting summary

Further information on research design is available in the Nature Research Reporting Summary linked to this article.

## Online content

Any methods, additional references, Nature Research reporting summaries, source data, extended data, supplementary information, acknowledgements, peer review information; details of author contributions and competing interests; and statements of data and code availability are available at 10.1038/s41589-022-01095-3.

## Supplementary information

Supplementary Information Supplementary Figs 1–15, Supplementary Tables 1–3.

Reporting Summary

Supplementary Data Supplementary data for Supplementary Figures

## Data Availability

DNA sequences are publicly available at benchling.com (https://benchling.com/tobstr/f_/zjVVwcqT-strittmatter-et-al-2022-natchembio-amber/) and at Genbank (ON681641–ON681702). Genbank accession numbers are provided with the plasmid table (ON681641–ON681702; Supplementary Table 1). Raw data for each figure is provided as .xlsx files alongside the manuscript. Source data are provided with this paper.

## Source data

Source Data Figure 1 Source data.
Source Data Figure 2 Source data.
Source Data Figure 3 Source data.
Source Data Figure 4 Source data.
Source Data Figure 5 Source data.
Source Data Figure 6 Source data.
Source Data Extended Data Figure 3 Source data.
Source Data Extended Data Figure 4 Source data.
Source Data Extended Data Figure 5 Source data.
Source Data Extended Data Figure 7 Source data.
