# Supplementary material for: Programmable DARPin-based receptors for the detection of thrombotic markers
Source: Nat Chem Biol. 2022 Aug 8;18(10):1125–34. doi: 10.1038/s41589-022-01095-3 (PMC9512699; doi:10.1038/s41589-022-01095-3)
Supplement: Supplementary file 1 — Supplementary Figs 1–15, Supplementary Tables 1–3. [file 41589_2022_1095_MOESM1_ESM.pdf]

nature portfolio

https://doi.org/10.1038/s41589-022-01095-3

Supplementary information

Programmable DARPin-based receptors for the detection of thrombotic markers

In the format provided by the authors and unedited

Supplementary Information

Programmable DARPin-based receptors for the detection of thrombotic markers

Tobias Strittmatter[1,2], Yidan Wang[3], Adrian Bertschi[1], Leo Scheller[1,4], Patrick C. Freitag[5], Preetam Guha Ray[1], Pascal Stuecheli[7], Jonas V. Schaefer[5,6], Thomas Reinberg[5], Dimitrios Tsakiris[8], Andreas Plückthun[5], Haifeng Ye[3], Martin Fussenegger[1,9]

[1]Department of Biosystems Science and Engineering, ETH Zurich, Basel, Switzerland.

[2]Present address: Roche Diagnostics GmbH, Penzberg, Germany

[3]Shanghai Key Laboratory of Regulatory Biology, Institute of Biomedical Sciences and School of Life Sciences, East China Normal University, Shanghai 200241, People's Republic of China.

[4]Present address: Laboratory of Protein Design & Immunoengineering, École Polytechnique Fédérale de Lausanne | EPFL, Lausanne, Switzerland

[5]Department of Biochemistry, University of Zurich, Zürich, Switzerland

[6]Present address: Novartis Institutes for BioMedical Research (NIBR), Basel, Switzerland

[7]Present address: Johnson & Johnson, Basel, Switzerland

[8]Diagnostic Hematology, University Hospital Basel, Basel, Switzerland

[9]University of Basel, Faculty of Science, Basel, Switzerland.

Supplementary Figure 1 - SDS-PAGE of proteins used

a) SDS-PAGE purified GFP-GFP fusions

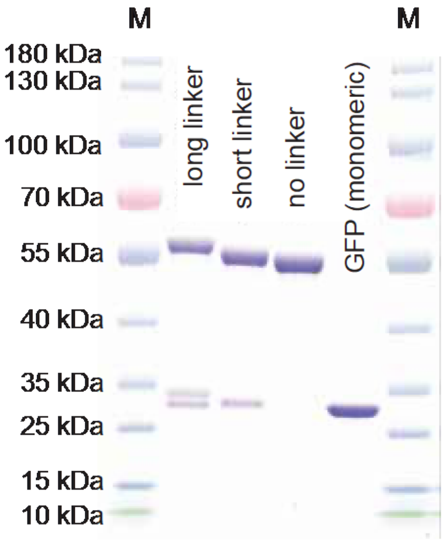

b) non reducing SDS-PAGE (6%) of xFDP preparation

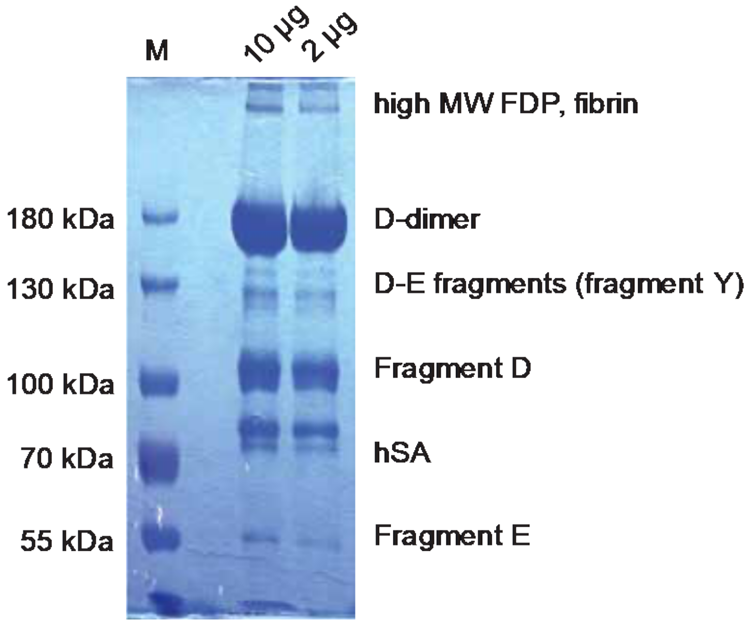

Supplementary Figure 1: a) Three GFP-GFP fusion proteins with various linker configurations, as well as monomeric GFP, were expressed as His-tag fusion proteins in *E. coli* and purified by Immobilized metal affinity chromatography (IMAC) followed by size

exclusion chromatography as detailed in the methods. Sequences are provided in Table S3. b) Result of non-reducing polyacrylamide gel-electrophoresis of the target protein (“native human D-dimer protein”, Abcam, ab98311) used in all experiments, done with a 6 % gel. Analysis suggests that the mixture consists primarily of D-dimer protein but contains significant amounts of other proteins. The putative identities of the other proteins are shown on the right-hand side. M: PageRuler Prestained Protein Ladder, 10 to 180 kDa. Pictures were produced from single experiments.

Supplementary Figure 2 - sequence analysis of candidate DARPins

a) Simple phylogenetic analysis of DARPin amino acid sequence

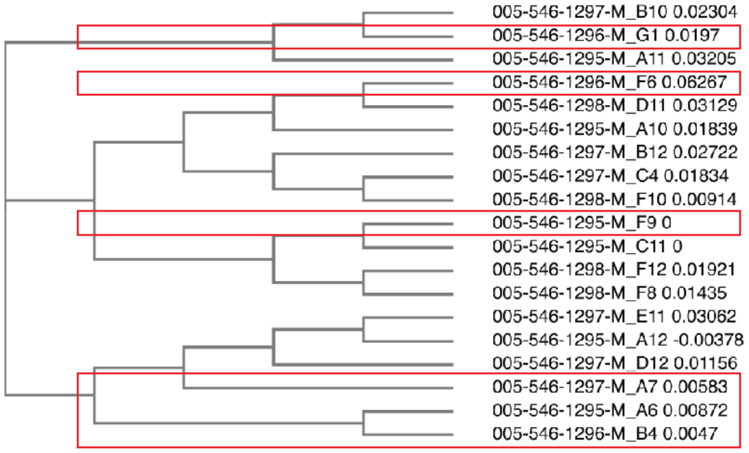

b) CLUSTAL O (1.2.4) multiple sequence alignment

```
                    Histag        N-cap                    1st repeat
005-546-1296-M_F6   MRGSHHHHHHHHGSDLGKKLLEAATSGQDDEVRILMANGADVNAMDHWGWTPLHLAAIEG 60
005-546-1295-M_F9   MRGSHHHHHHHHGSDLGKKLLEAARAGQDDEVRILMANGADVNAQDIWGWTPLHLAAYAG 60
005-546-1296-M_G1   MRGSHHHHHHHHGSDLGKKLLEAARAGQDDEVRILMANGADVNA---------------- 45
005-546-1297-M_A7   MRGSHHHHHHHHGSDLGKKLLEAARAGQDDEVRILMANGADVNA---------------- 45
005-546-1295-M_A6   MRGSHHHHHHHHGSDLGKKLLEAARAGQDDEVRILMANGADVNA---------------- 45
005-546-1296-M_B4   MRGSHHHHHHHHGSDLGKKLLEAARAGQDDEVRILMANGADVNA---------------- 45
                    ************************ :*******************

                                            2nd repeat
005-546-1296-M_F6   HQEIVEVLLKTGADVNAKDQWGATPLHLAAVVGHLEIVEVLLKHGADVNA---------- 98
005-546-1295-M_F9   HLEIVKVLLKTGADVNAYDDWGSTPLHLAAWIGHLEIVEVLLKAGADVNAWDVHGFTPLH 120
005-546-1296-M_G1   -----------------TDNWGDTPLHLAAWHGHLEIVEVLLKTGADVNAQDIIGATPLH 87
005-546-1297-M_A7   -----------------FDWYGTTPLHLAAFNGHLEIVEVLLKTGADVNAQDLFGNTPLH 87
005-546-1295-M_A6   -----------------EDWYGTTPLHLAAHNGHLEIVEVLLKTGADVNAQDLFGNTPLH 87
005-546-1296-M_B4   -----------------EDWYGTTPLHLAAHNGHLEIVEVLLKTGADVNAQDLFGNTPLH 87
                                     * :* *******  ******************

                       3rd repeat             C-cap
005-546-1296-M_F6   -----------------------QDISGQTPFDLAAWHGNEDIAEVLQKAAKLNDYKDDD 147
005-546-1295-M_F9   LAAIRGHLEIVEVLLKHGADVNAQDKFGKTPFDLAIDNGNEDIAEVLQKAAKLNDYKDDD 180
005-546-1296-M_G1   LAAIMGHLEIVEVLLKAGADVNAQDKFGKTPFDLAIDNGNEDIAEVLQKAAKLNDYKDDD 147
005-546-1297-M_A7   LAAWNGHLEIVEVLLKHGADVNAQDKFGKTPFDLAIDNGNEDIAEVLQKAAKLNDYKDDD 147
005-546-1295-M_A6   LAAYEGHLEIVEVLLKHGADVNAQDKFGKTPFDLAIDNGNEDIAEVLQKAAKLNDYKDDD 147
005-546-1296-M_B4   LAAWNGHLEIVEVLLKHGADVNAQDKFGKTPFDLAIDNGNEDIAEVLQKAAKLNDYKDDD 147
                                           **   *:******   :*********************


005-546-1296-M_F6   DK*   149
005-546-1295-M_F9   DK*   182
005-546-1296-M_G1   DK*   149
005-546-1297-M_A7   DK*   149
005-546-1295-M_A6   DK*   149
005-546-1296-M_B4   DK*   149
                    ***
```

	identity	similarity
F6	86.0 %	90.0 %
F9	74.9 %	78.7 %
G1	92.0 %	94.0 %
A7	97.3 %	98.0 %
A6	100.0 %	100.0 %
B4	98.7 %	99.3 %

Supplementary Figure 2: Characterization of DARPin binders. a) Simple phylogenetic analysis of all DARPins based on their amino acid sequences. DARPins G1, F6, F9, A7, A6 and B4 are boxed in red. DARPins G1, F6, F9, A7, A6 and B4 were further analyzed by b) alignment of amino acid sequences using the Clustal Omega 1.2.4 algorithm and c) similarity analysis based on pairwise alignments employing the EMBOSS-needle algorithm.

Supplementary Figure 3 - oligomerization analysis of candidate DARPins

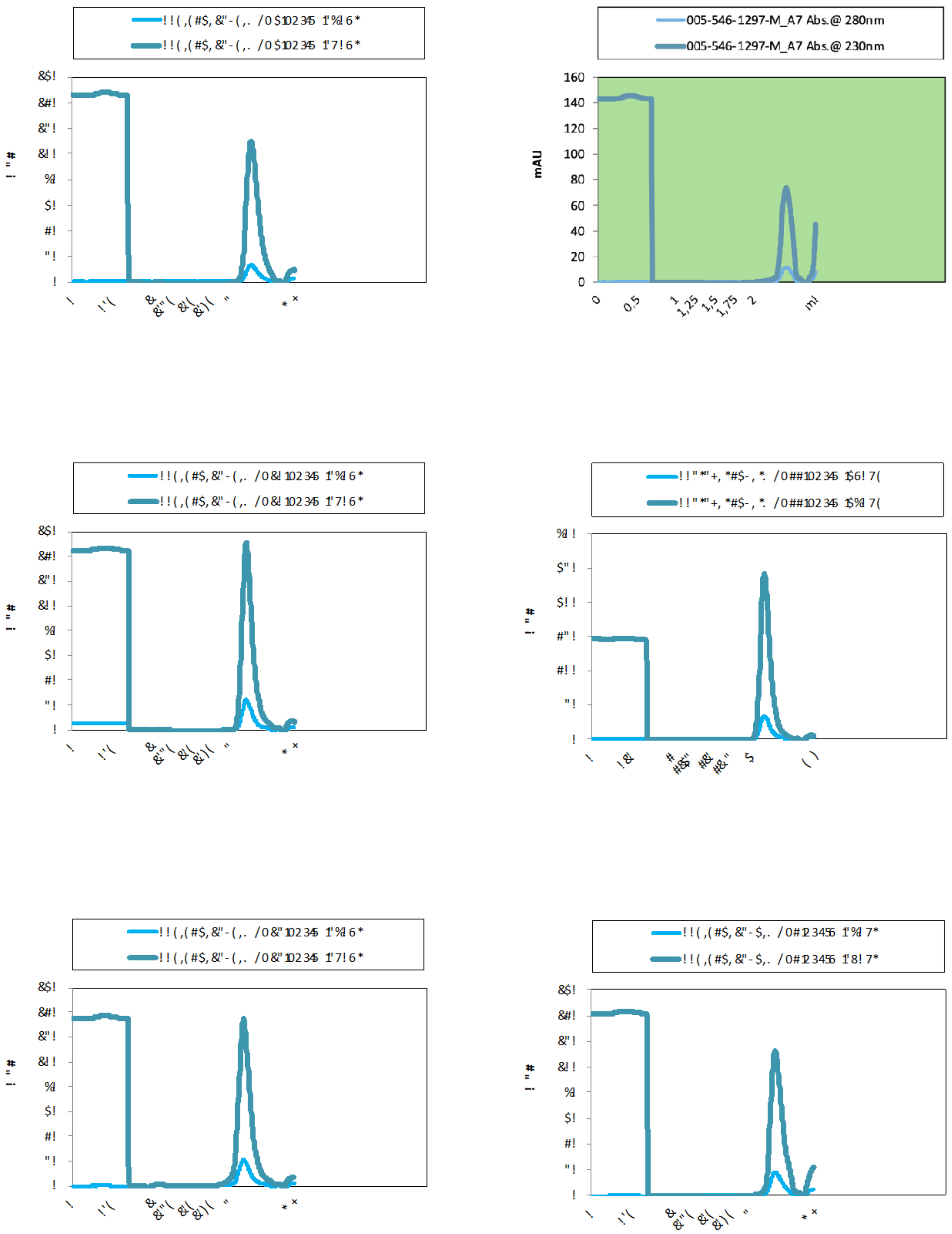

Supplementary Figure 3: Size exclusion chromatography was performed to assess the dimerization behavior of selected DARPins. Chromatograms in green were found to

represent monomeric candidates. Chromatograms were produced from single runs of each binder and were not reproduced.

Supplementary Figure 3 continued - oligomerization analysis of candidate DARPins

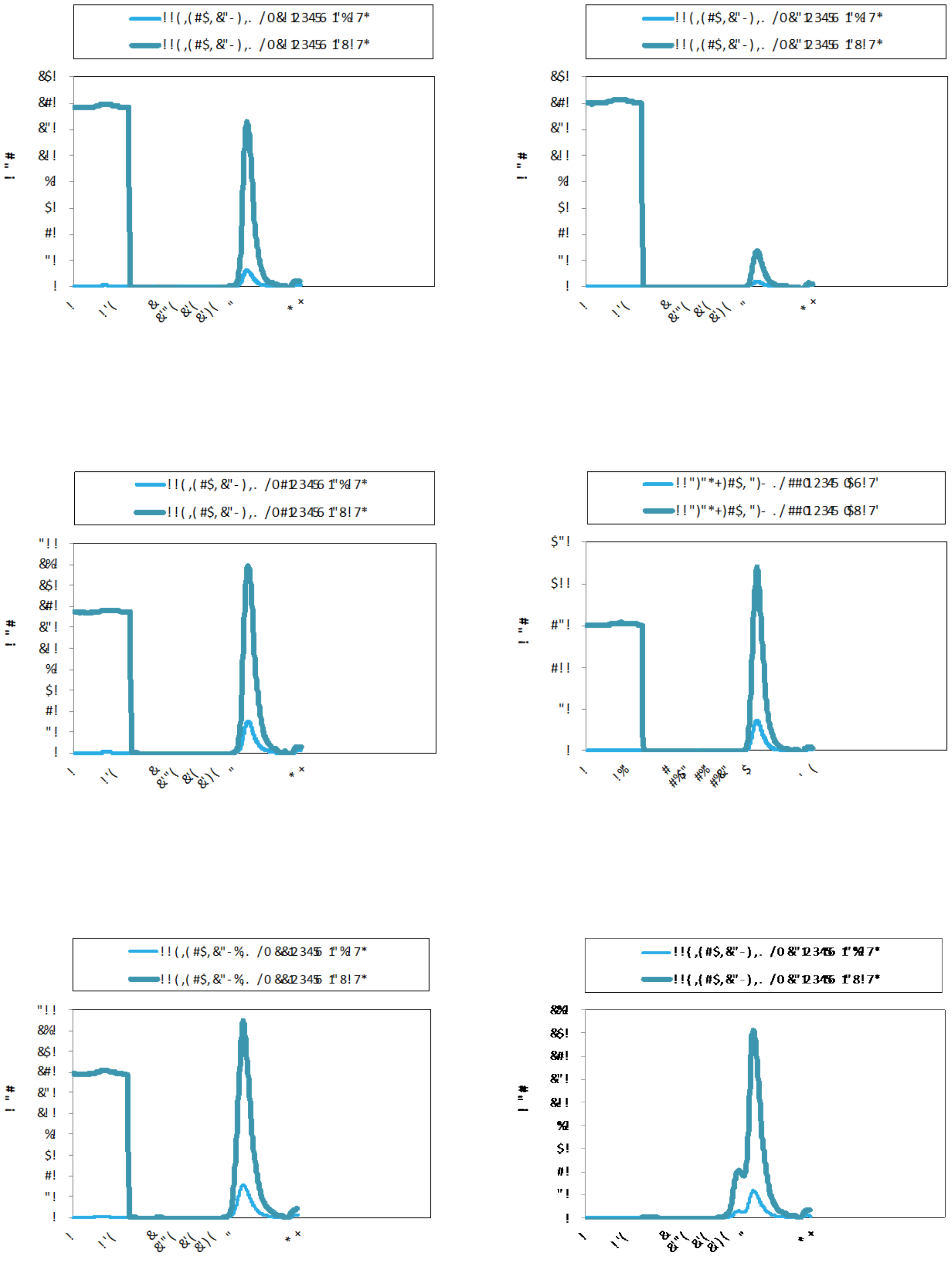

Supplementary Figure 3: part2, continued.

Supplementary Figure 3 continued - oligomerization analysis of candidate DARPins

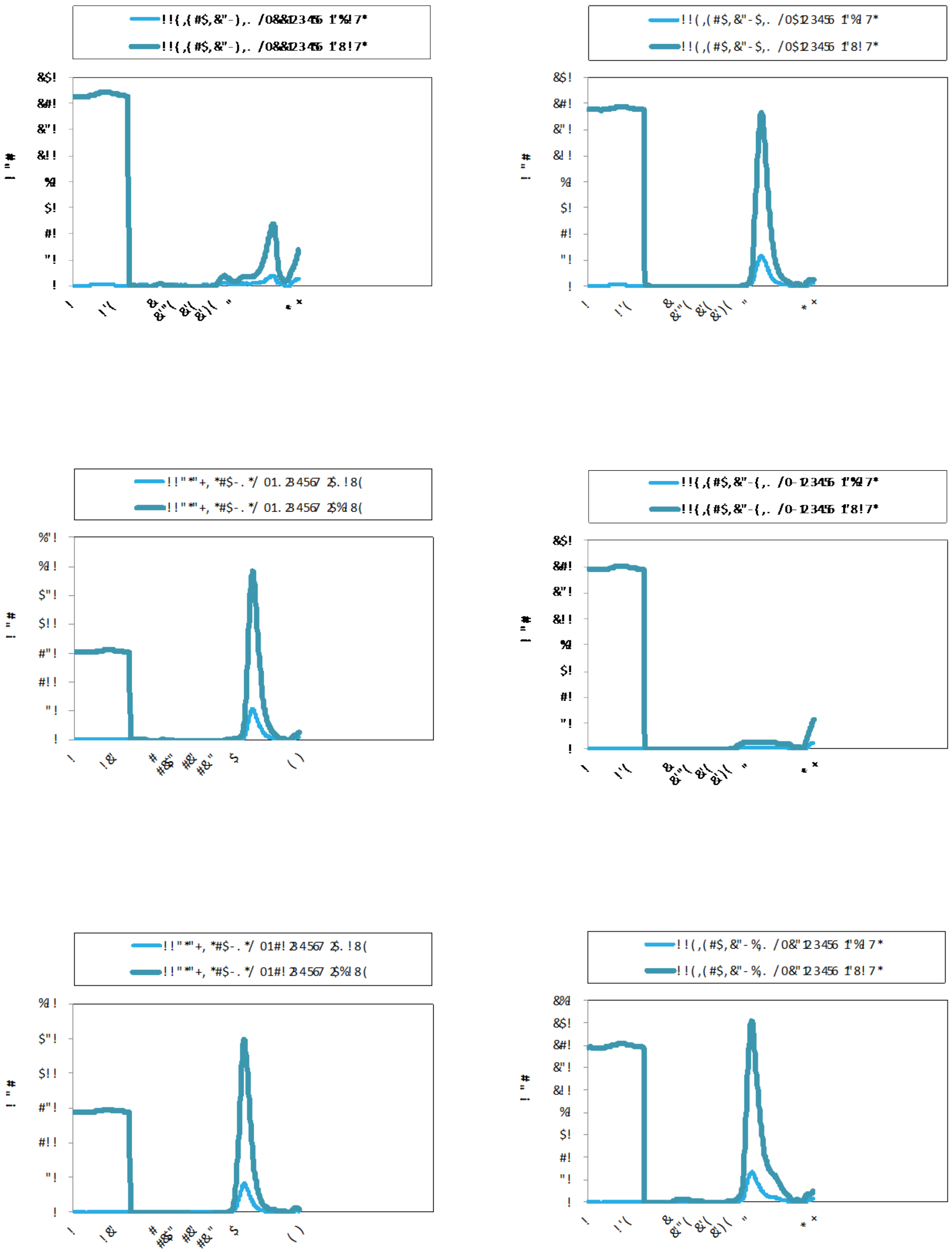

Supplementary Figure 3: part3, continued.

Supplementary Figure 3 continued - oligomerization analysis of candidate DARPins

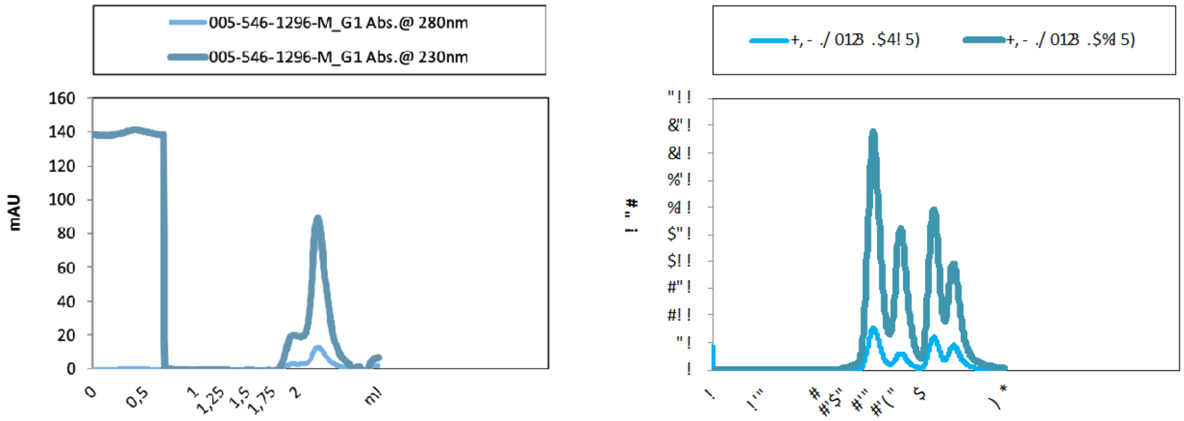

Supplementary Figure 3: part4, continued.

Supplementary Figure 4 - toxicity and specificity controls

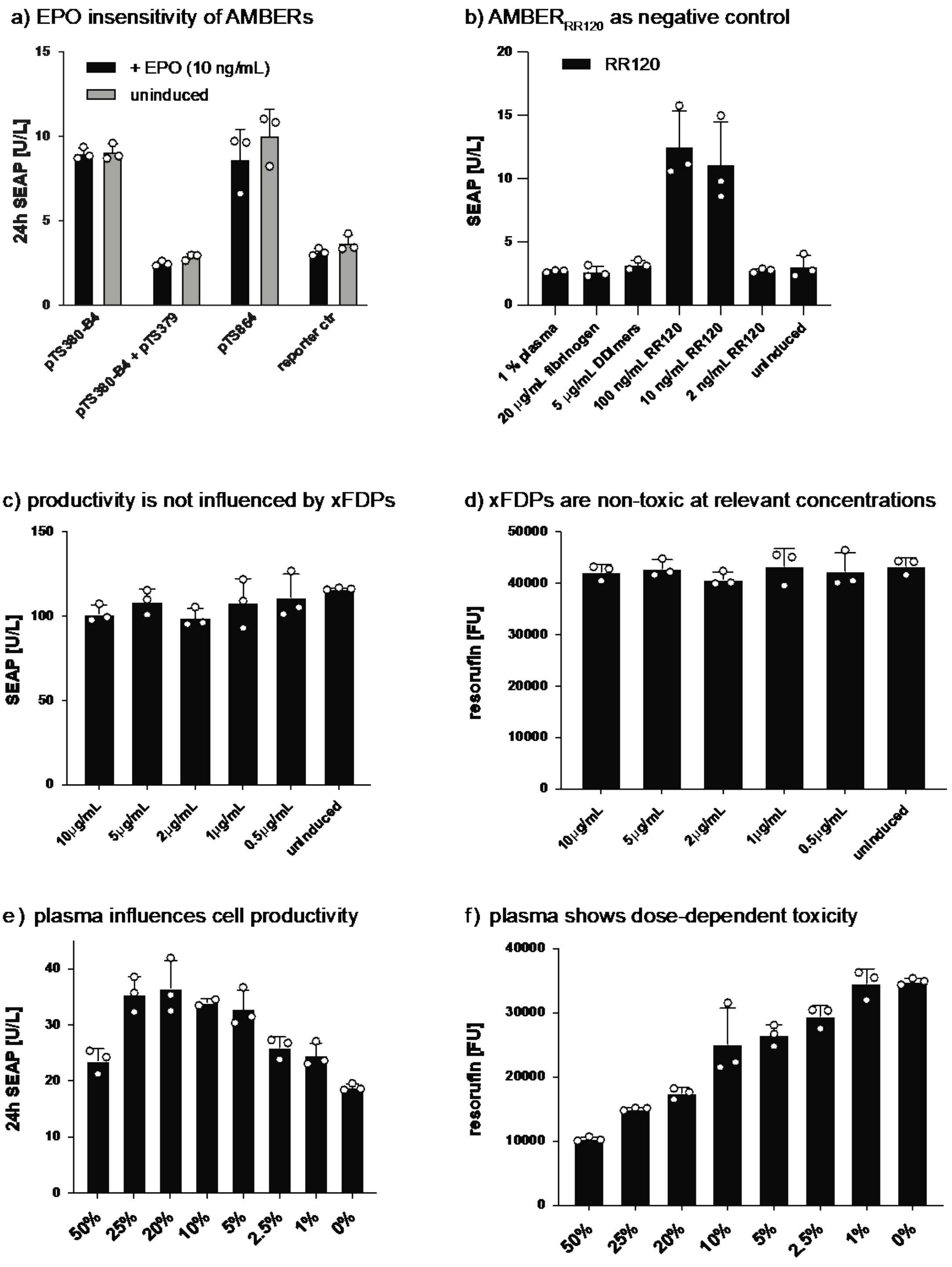

Supplementary Figure 4: Control experiments to assess the cross-reactivity of receptors and inputs, as well as the toxicity of inducers. HEK-293T cells were transfected overnight with the indicated receptors and the pLS13 STAT3 reporter plasmid alongside pLS15 for STAT3 transcription factor overexpression or a constitutive SEAP-expressing plasmid (pSEAP2ctr),

as described in the methods. a) None of the receptor scaffolds used is responsive to erythropoetin (EPO), which is the natural ligand of the native EPO receptor. Also, the STAT3 pathway is not induced by EPO administration. Cells were incubated with 10 ng/mL EPO protein in complete DMEM containing 10 % FCS for 24 h and the supernatant was sampled for analysis of SEAP reporter activity. b) The RR120 receptor used as the negative control exclusively responds to its ligand RR120 and is not activated by plasma, fibrinogen or xFDPs. c-f) Productivity and viability of HEK-293T cells were assessed in c) and e) in terms of constitutive expression of SEAP and in d) and f) by measuring resazurin reduction, which leads to production of the fluorescent dye resorufin, in parallel. HEK-293T cells were transfected overnight with pSEAP2ctr prior to incubation with c)-d) xFDPs or e)-f) plasma at the indicated concentrations. At 24 h after induction, the supernatant was sampled and SEAP activity was measured. After sampling, the medium was exchanged for 100 µL of complete DMEM containing 10 % FCS and resazurin. Cells were incubated with resazurin for approximately 30 minutes and 60 µL of medium was transferred to a clear-bottomed assay plate for fluorescence recording. All values are means ± SD of n = 3 independent samples.

Supplementary Figure 5 - Screening of dimeric receptors

a)

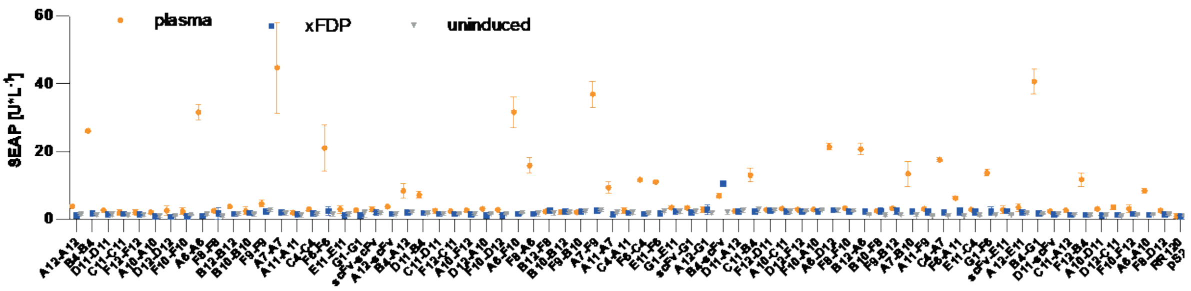

b)

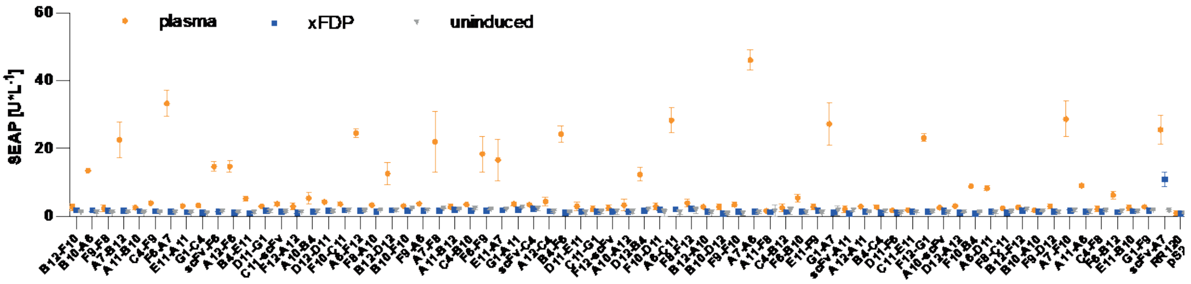

c)

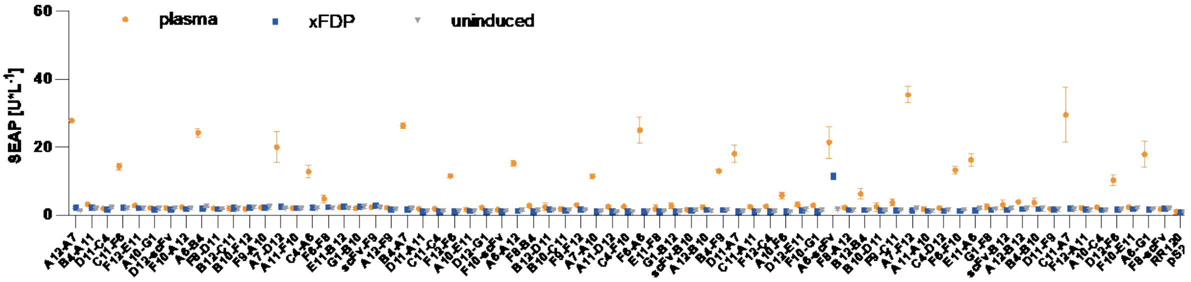

Supplementary Figure 5: Screening of homodimeric receptors in HEK-293T. Cells were incubated with 0.5 µg/mL xFDPs or 0.5 % (v/v) reconstituted human plasma in complete DMEM containing 10 % FCS for 24 h, and SEAP reporter activity was measured. Subsets consisting of 70 combinations each. a) 1-70, b) 71-140, c) 141-210. All panels include RR120 as a negative control and constitutive expression of SEAP as a reference. All values are means ± SD of n = 3 independent samples.

Supplementary Figure 6 - correlation of HTRF with likelihood to find functional receptors

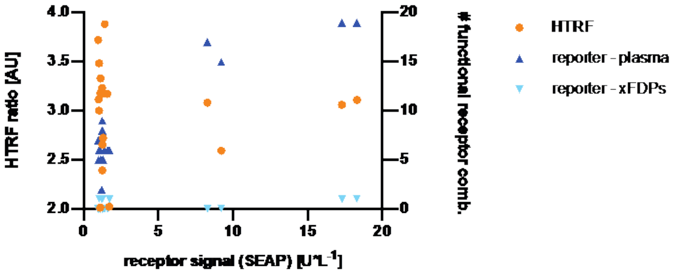

Supplementary Figure 6: Correlation of receptor activation by plasma of DARPin-AMBERs in homodimeric configuration measured in terms of SEAP reporter expression with the corresponding signal intensities from HTRF (left y-axis), as well as the number of functional receptor combinations (fold induction >5) for coagulation or xFDP detection (right y-axis). HTRF signals are single values while receptor activation is presented as means of n = 3 independent samples.

Supplementary Figure 7 - screening of tandem receptors

a) DARPin-based tandem receptors induced with plasma and xFDPs

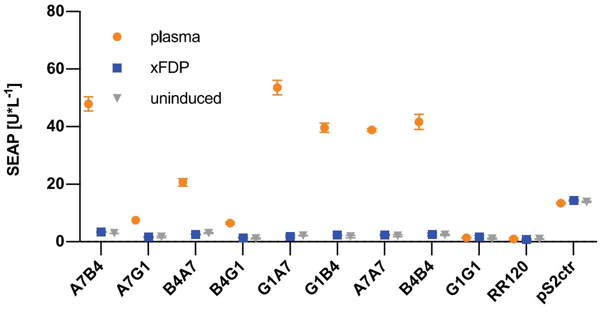

b) fold induction of DARPin-based tandem receptors induced with plasma and xFDPs

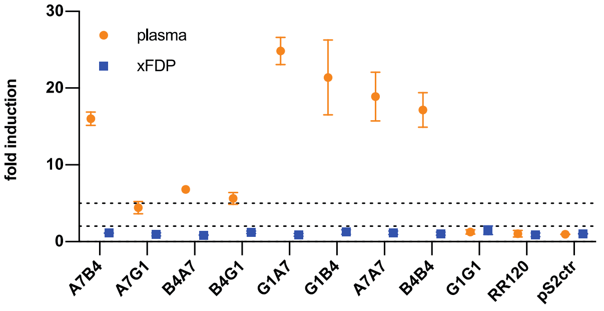

c) scF_v-based tandem receptors induced with plasma and xFDPs

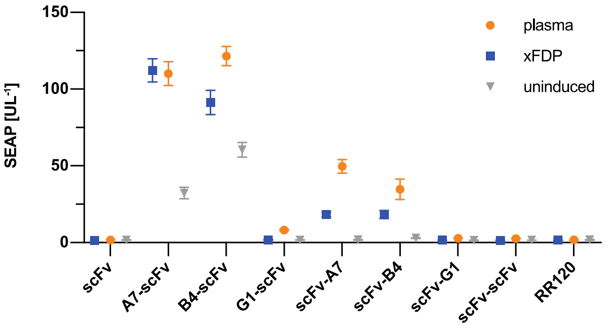

d) fold induction of scF_v-based tandem receptors induced with plasma and xFDPs

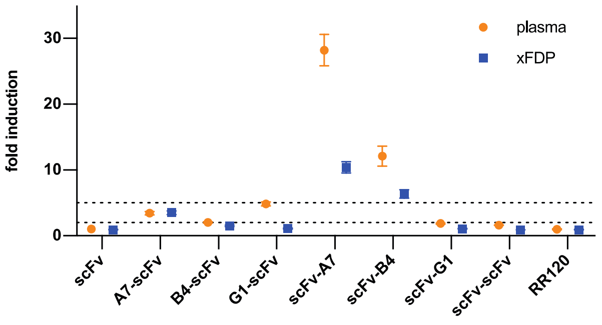

Supplementary Figure 7: Screening of tandem receptors based on DARPins only (a-b) or scFv-DARPin combinations (c-d). HEK-293T cells were transfected overnight with the

indicated receptors and the pLS13 STAT3 reporter plasmid alongside pLS15 for STAT3 transcription factor overexpression, as described in the methods. a) and c) Cells were incubated for 24 h in complete DMEM containing 10 % FCS and xFDPs at 0.5 µg/mL or plasma at a final concentration of 0.5 % (v/v) prior to assessment of SEAP reporter activity. b) and d) Fold induction was calculated for each receptor by normalizing samples induced with plasma or xFDP to uninduced control samples. All values are means ± SD of n = 3 independent samples.

Supplementary Figure 8 - Electrochemical impedence spectroscopy (EIS)

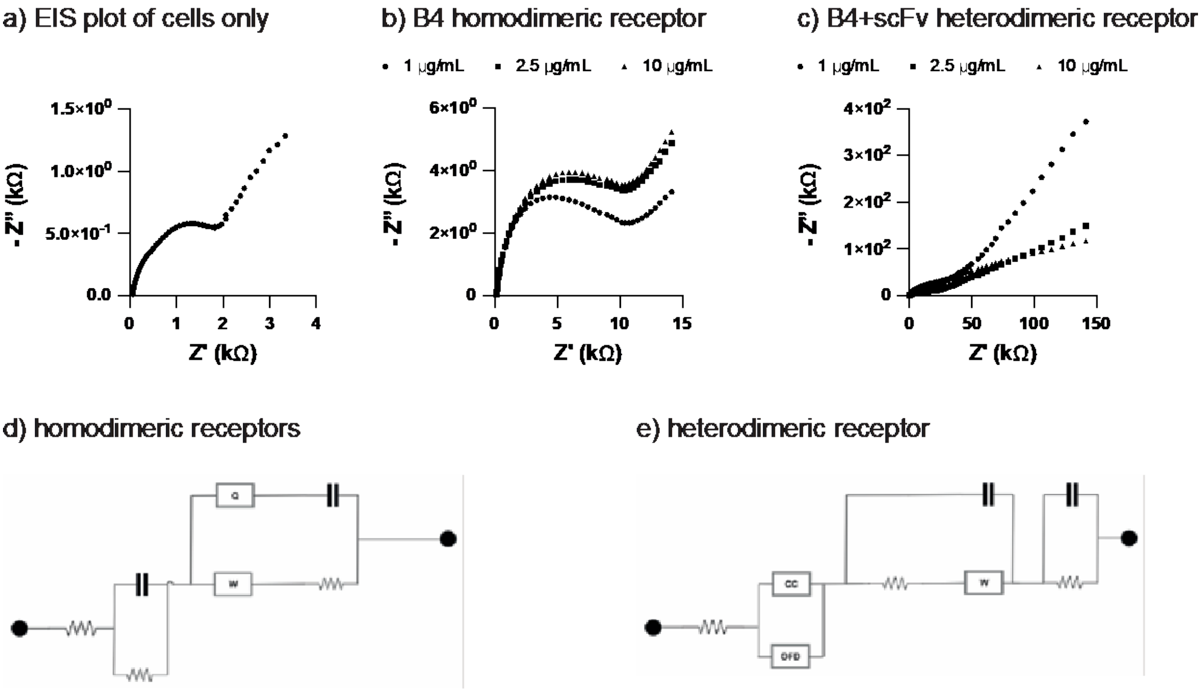

Supplementary Figure 8: Plots of EIS measurements to measure resistance in response to varying concentrations of xFDPs for a) The background signal of only cells is low compared to b) the B4 homodimeric receptor and the c) B4+scFv heterodimeric receptor exposed to xFDPs. Equivalent circuits used to calculate the charge transfer resistance (R_{ct}) for d) homodimeric receptors and e) heterodimeric receptors. Two different circuits were used due to differences in signal intensity. Q: parallel constant phase element, W: Warburg impedance; CC: Cole-Cole element; OFD: open finite diffusion element; parallel lines: double layer capacitor; zigzag: resistor. Values in a-c) are single measurements that have not been repeated.

Supplementary Figure 9 - confirming fibrinogen as an inducer molecule

a) activity analysis of size exclusion chromatography derived fractions

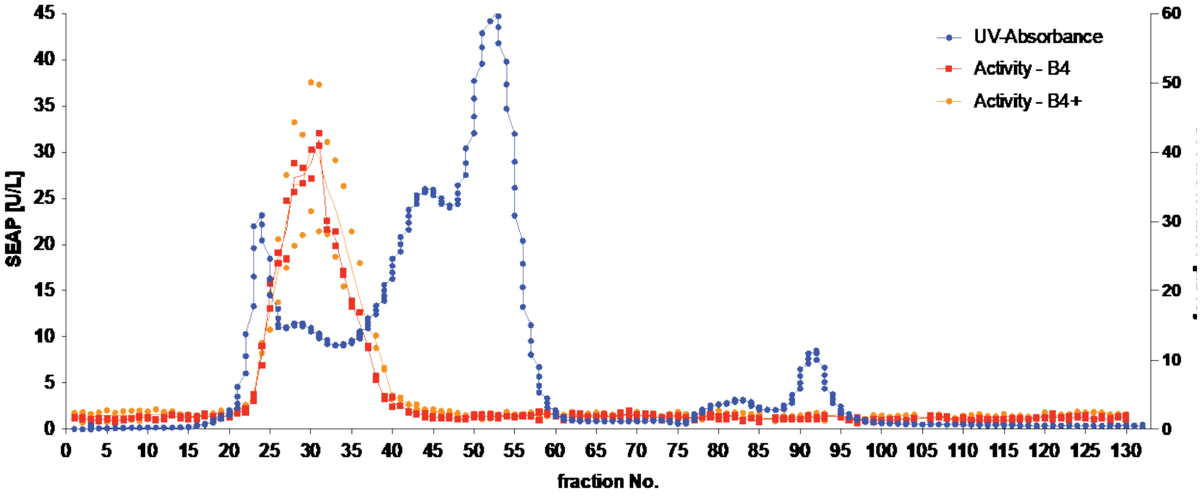

b) SDS-PAGE and mass-spectrometric analysis of relevant fractions

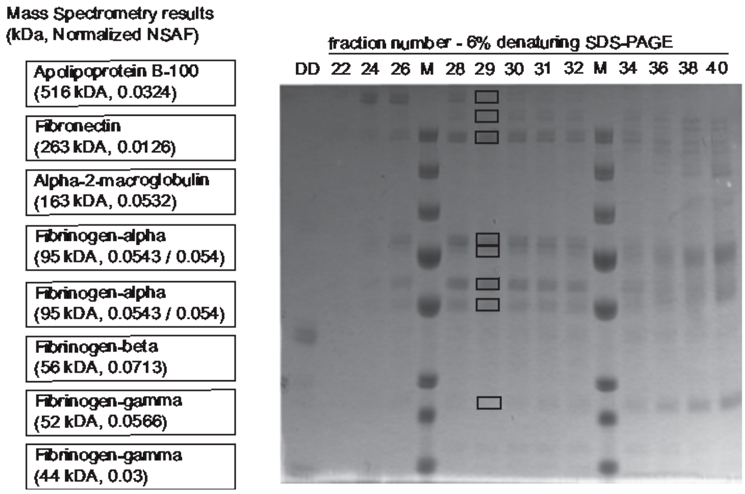

Supplementary Figure 9: Analysis of plasma fractionated by size-exclusion chromatography. Size-exclusion chromatography (SEC) with a self-packed Sephadex 200 column was used to assess the active fraction of whole human plasma. Plasma was filtered through a 0.2 µm filter to remove most of the lipoprotein vesicles prior to loading. a) UV detection of SEC experiment (blue) correlated with the activity of each fraction in the cell culture (red and orange). b) SDS-PAGE under denaturing conditions of relevant fractions from the SEC experiment. Boxed bands were excised and subjected to mass spectrometry for identification. The results of mass spectrometry are presented in boxes on the left-hand side, including the molecular mass of the respective full-length protein along with the relative abundance of the indicated protein in the sample. Dots represent in a) are single values, lines represent the mean.

Supplementary Figure 10 - fragments D and E of fibrinogen

a) fragment D of fibrinogen

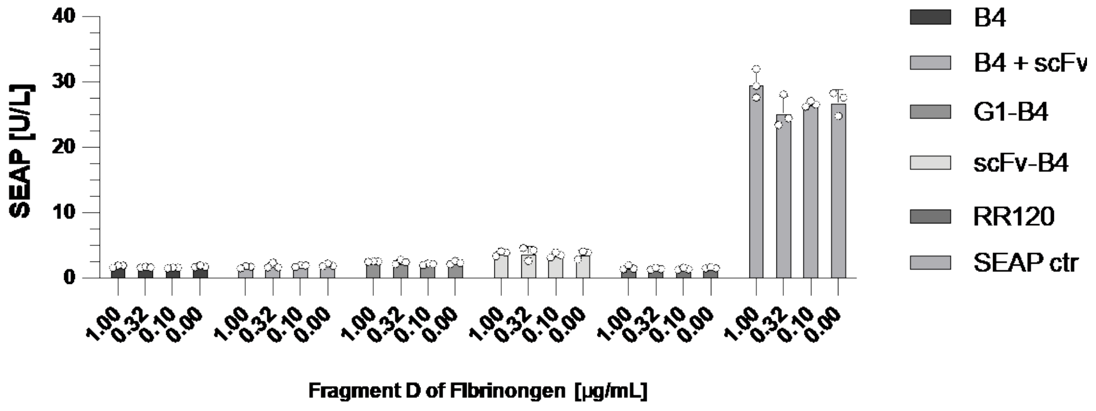

b) fragment E of fibrinogen

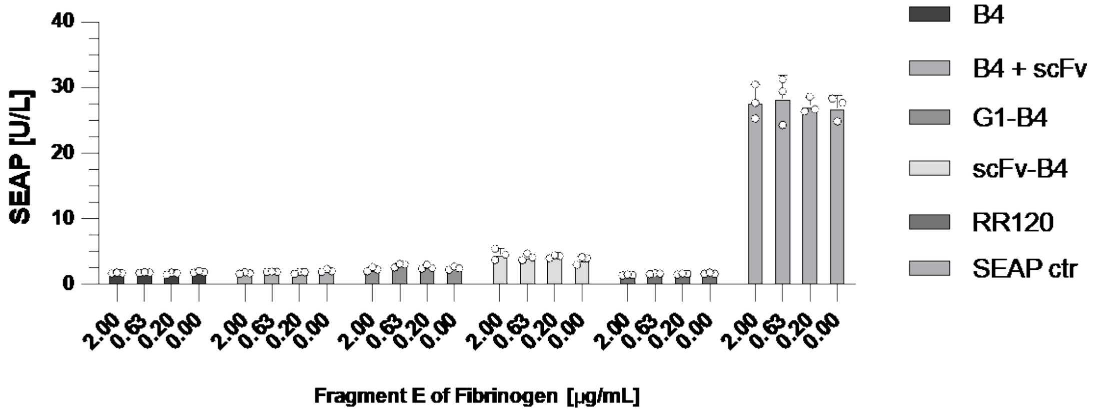

c) mixture of fragments D and E of fibrinogen

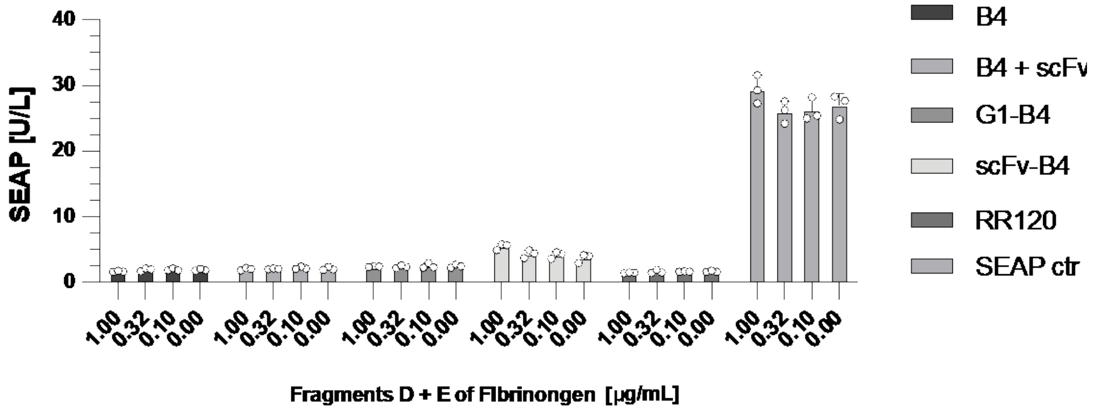

Supplementary Figure 10: Activation of receptors by plasmin degradation products of native fibrinogen was assessed. Cells were incubated for 24 h in complete DMEM containing 10 % FCS and a) purified fragment D protein of fibrinogen at 1, 0.32, 0.1 or 0 µg/mL or b) purified fragment E protein of fibrinogen at 2, 0.63, 0.2 or 0 µg/mL or c) a combination of both fragments D and E of fibrinogen at the indicated concentrations. All values are means ± SD of n = 3 independent samples.

Supplementary Figure 11 - plasmin on fibrinogen

a) plasmin degrades fibrinogen at high concentrations

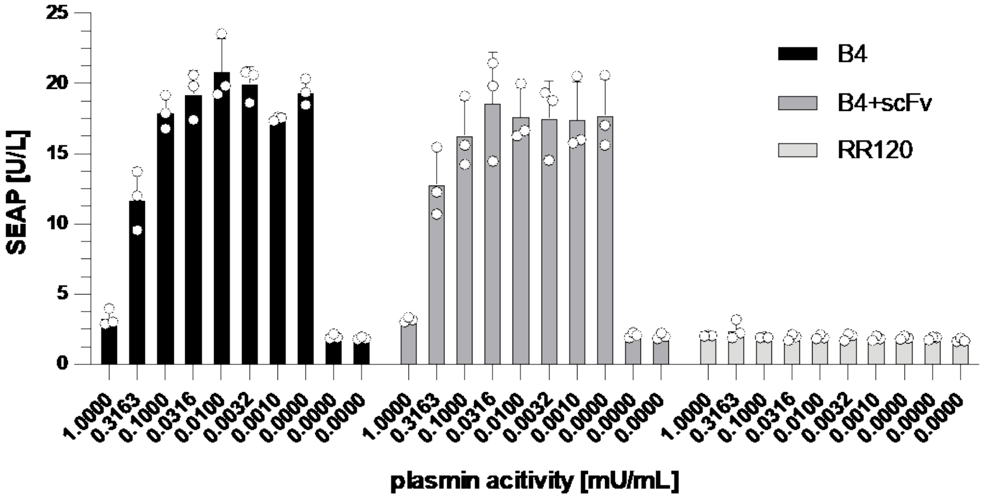

b) plasmin on fibrinogen

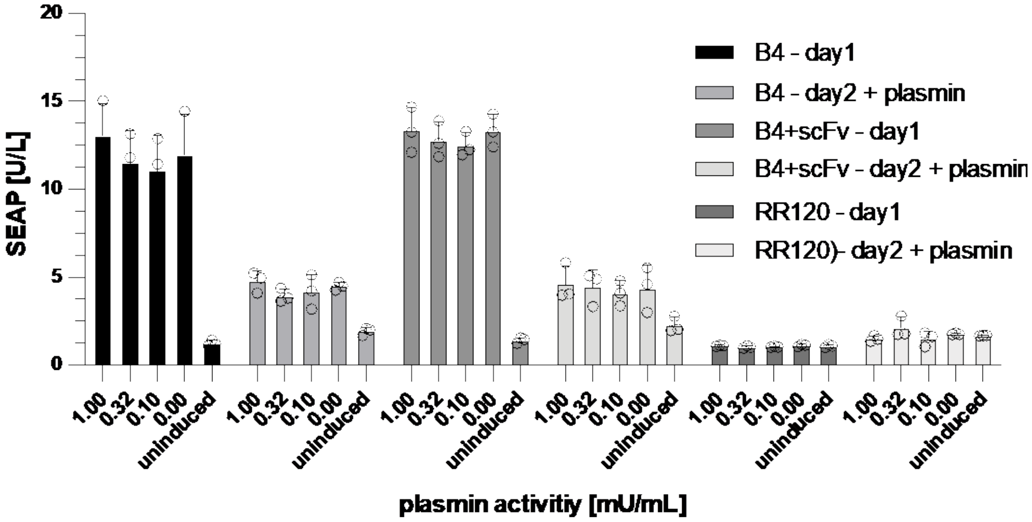

c) plasmin on xFDPs

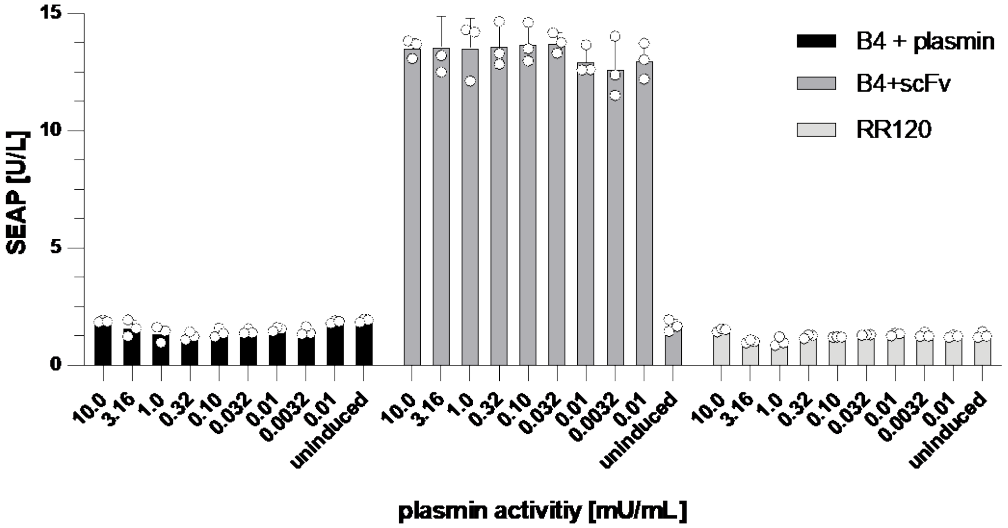

Supplementary Figure 11: Additional characterization of inducer molecules. Cells were incubated for 24 h in complete DMEM containing 10 % FCS and the test substance. a) Fibrinogen-mediated activity of $AMBER_{B4}$, $AMBER_{B4/scFv}$ or $AMBER_{G1\text{-}B4}$ is reduced by high activity of plasmin at 1 mU/mL. Fibrinogen was incubated at 5 µg/mL together with the indicated concentrations of plasmin. Sensitivity to plasmin depends on the receptor configuration. b) Cells expressing $AMBER_{B4}$ or $AMBER_{B4/scFv}$ or RR120 receptor were activated with fibrinogen at 5 µg/mL for 24 h prior to replacing the medium with complete DMEM containing no fibrinogen, but with the indicated activity of plasmin instead. No difference in activation can be observed between different concentrations of plasmin. c) xFDP-activated expression of SEAP reporter is independent of plasmin activity in the supernatant. Cells were incubated with 1 µg/mL xFDP protein and supplemented with the indicated plasmin activity. All values are means ± SD of n = 3 independent samples.

Supplementary Figure 12 - specificity assays

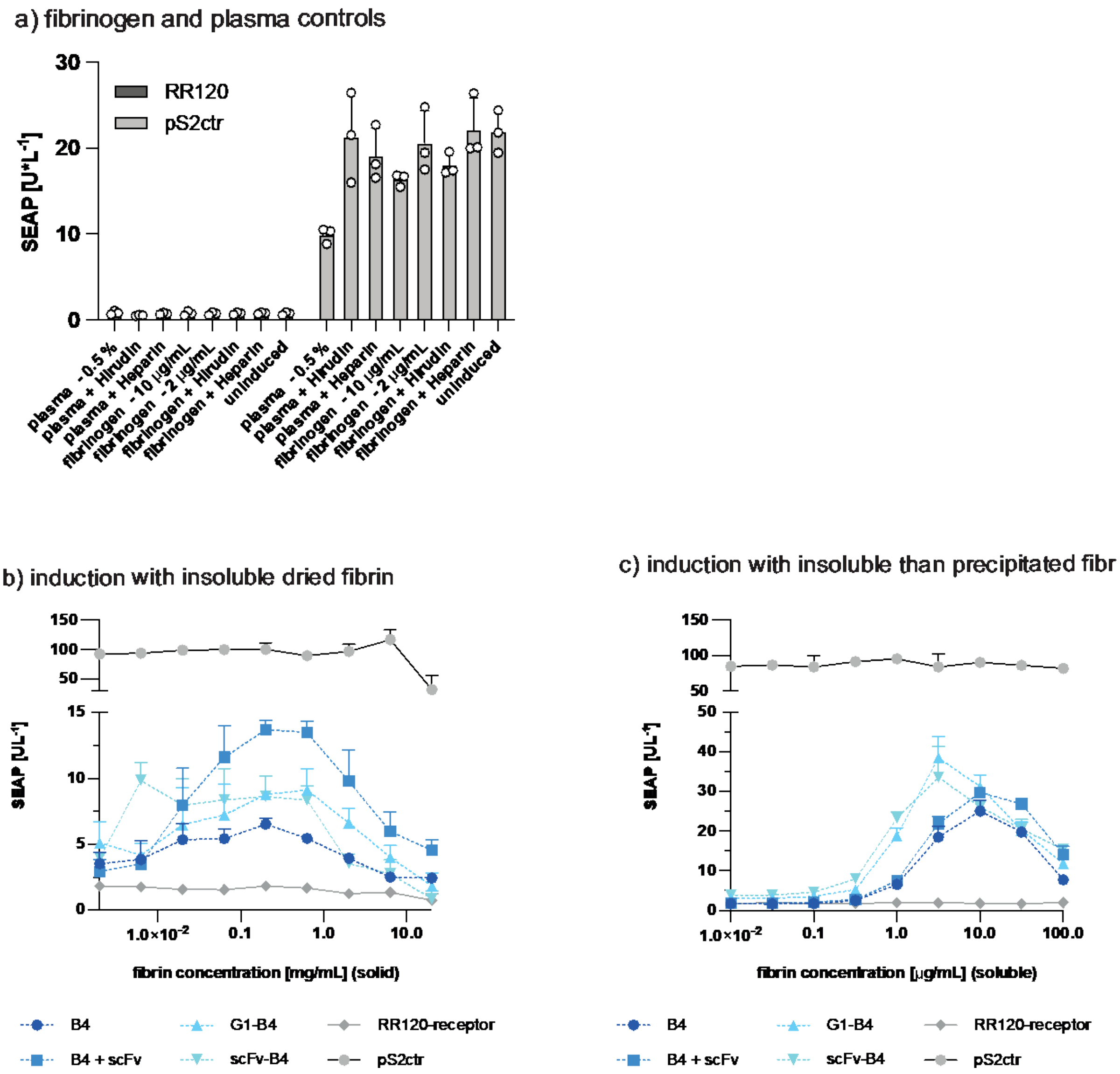

Supplementary Figure 12: a) Unspecific activation of receptors under the conditions used in figure 4b was excluded by measuring the responses obtained with RR120-receptor-transfected HEK-293T cells harboring the pLS13 STAT3 reporter plasmid and pLS15 for STAT3 transcription factor overexpression. A dose-response plot of fibrin on $AMBER_{B4}$, $AMBER_{B4/scFv}$, $AMBER_{G1\text{-}B4}$ or $AMBER_{scFv\text{-}B4}$ at 10 dose levels revealed bell-shape-like activation patterns for b) an insoluble fibrin preparation and c) a solubilized fibrin preparation that becomes insoluble at neutral pH. All values are means ± SD of n = 3 independent samples.

Supplementary Figure 13 - control experiments for FXIII, plasmin, TXA and tridegi

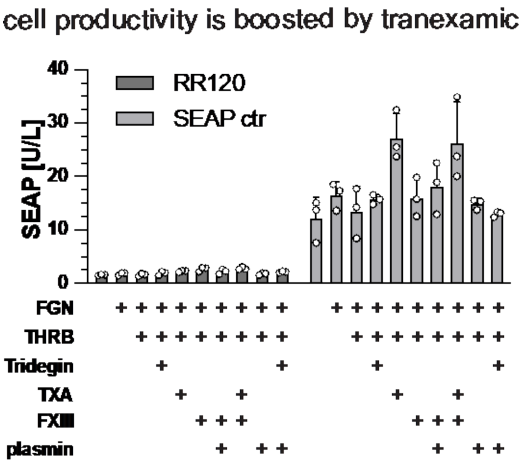

Supplementary Figure 13: Reference samples for assessment of cross-linking and fibrinolysis-modulating factors depicted in fig. 4c. HEK-293T cells were transfected with either the RR120 receptor in combination with pLS13 and pLS15 or pSEAP2ctr constitutively expressing SEAP reporter. The latter showed an unspecific boost of cell productivity in DMEM supplemented with 10 mg/mL TXA. All values are means ± SD of n = 3 independent samples.

Supplementary Figure 14 - stable cell line characterization

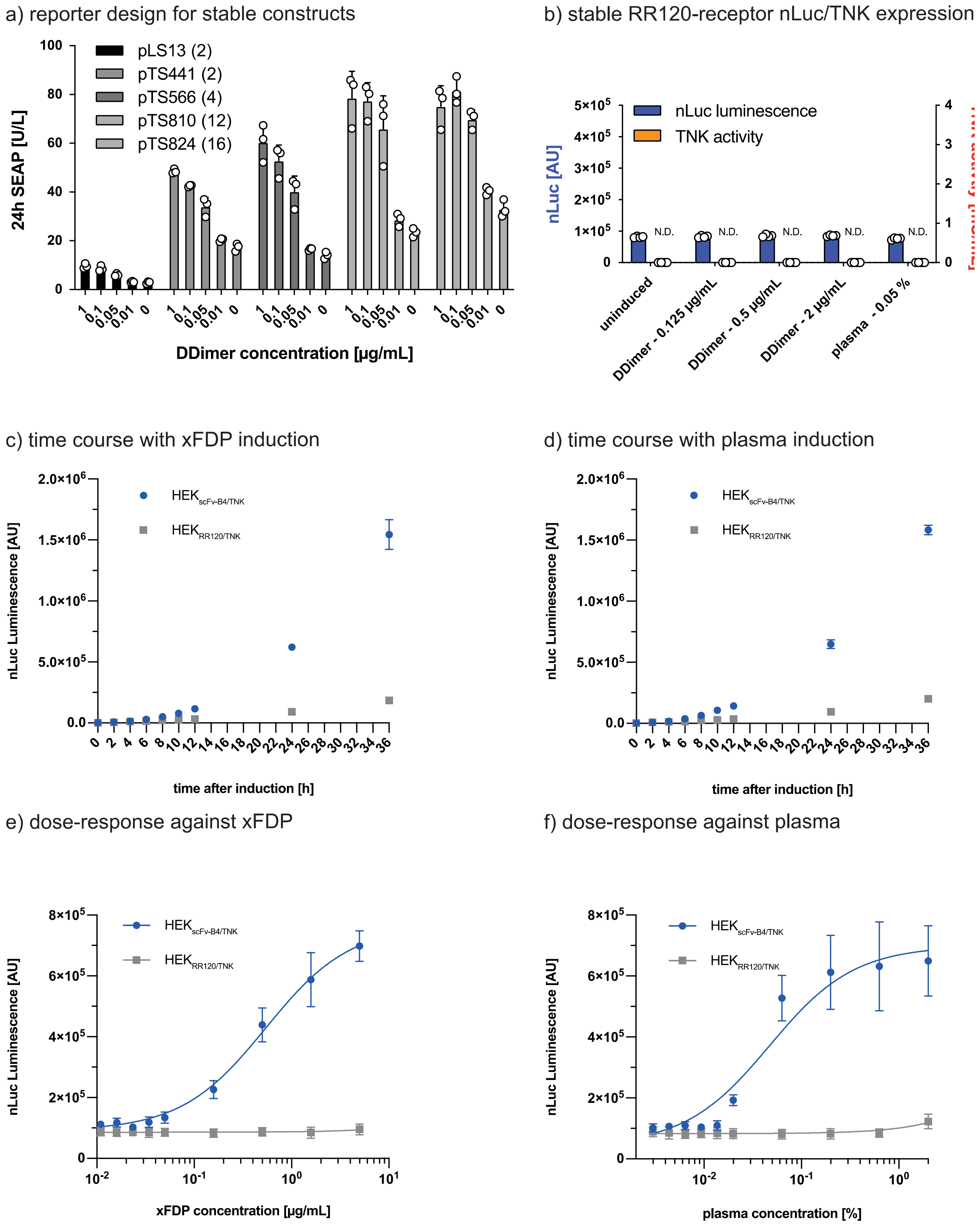

Supplementary Figure 14: Characterization of stable cell lines. a) Different reporter constructs were evaluated for their inducibility with xFDPs in combination with AMBER$_{B4/scFv}$. HEK-293T cells were transfected overnight with the indicated receptors and the pLS13 STAT3 reporter plasmid alongside pLS15 for STAT3 transcription factor

overexpression, as described in the methods. Cells were incubated for 24 h in complete DMEM containing 10 % FCS and the test substance. xFDPs were supplemented at the indicated concentrations, ranging from 1 µg/mL to 0.01 µg/mL. b) The stable polyclonal cell line $HEK_{RR120/TNK}$ bears the inactive RR120 receptor controlling a STAT3-driven reporter construct expressing reporter nano-luciferase (Nluc) coupled via a furin cleavage site to the therapeutic protein tenecteplase (TNK). 45,000 cells were seeded per well and incubation was done for 24 h. We subsequently induced these cells for 24 h with the indicated concentrations of xFDPs or plasma. Nluc and TNK activity were measured in the supernatant. The TNK activity of untreated cells was subtracted from the observed values. c)-f) Stable monoclonal $HEK_{scFv\text{-}B4/TNK}$ cells and polyclonal $HEK_{RR120/TNK}$ were seeded at 15,000 cells per well and induced 24 h after seeding. c)-d) Time courses of reporter gene expression after induction with either c) 1 µg/mL xFDPs or d) 1 % (v/v) reconstituted human plasma. Reporter gene activity was measured at the indicated time points post induction. e)-f) Dose responses of $HEK_{scFv\text{-}B4/TNK}$ and $HEK_{RR120/TNK}$ upon incubation with the indicated amounts of inducer. A $\sqrt{10}$-dilution series was prepared with e) 5 µg/mL to 0.011 µg/mL xFDPs. In f) stable cells were induced with plasma ranging from 2 % to 0.003 % (v/v). Values in a) are means ± SD of triplicate determinations, values in b) are means ± SD of n = 4 independent samples, values in c-f) are cumulative values of three independent measurements performed in triplicate, N = 9, shown as mean ± SD.

Supplementary Figure 15 - mouse vs human plasma

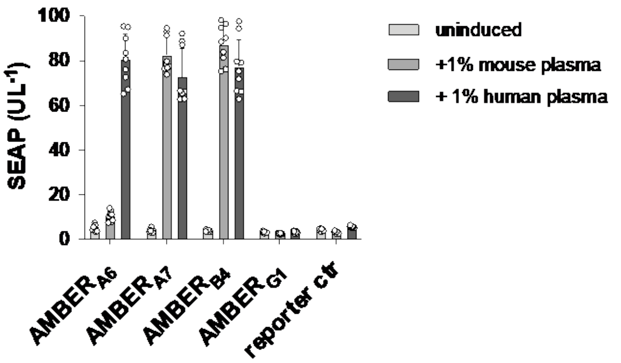

Supplementary Figure 15: Inducibility of (x)FDP sensing AMBERs by mouse plasma. HEK-293T cells were transfected overnight with the indicated receptors and the pLS13 STAT3 reporter plasmid alongside pLS15 for STAT3 transcription factor overexpression. The cells were incubated for 24 h in complete DMEM containing 10 % FCS and 1% (v/v) of either human or mouse plasma. Values are means ± SD of n = 9 independent samples.

Supplementary Table 1: Plasmid used in this work

All sequences are publicly available at Benchling.com: https://benchling.com/tobstr/f_/zjVVwcqT-strittmatter-et-al-2022-natchembio-amber/.

Plasmid Name	Description	Reference / GenBank No
005-546-1295-M_A6	DARPin DNA sequence extracted from bacterial expression plasmid bearing an inducible promoter for expression of DARPin-A6	This work / ON681641
005-546-1297-M_A7	DARPin DNA sequence extracted from bacterial expression plasmid bearing an inducible promoter for expression of DARPin-A7	This work / ON681642
005-546-1295-M_A10	DARPin DNA sequence extracted from bacterial expression plasmid bearing an inducible promoter for expression of DARPin-A10	This work / ON681643
005-546-1295-M_A11	DARPin DNA sequence extracted from bacterial expression plasmid bearing an inducible promoter for expression of DARPin-A11	This work / ON681644
005-546-1295-M_A12	DARPin DNA sequence extracted from bacterial expression plasmid bearing an inducible promoter for expression of DARPin-A12	This work / ON681645
005-546-1296-M_B4	DARPin DNA sequence extracted from bacterial expression plasmid bearing an inducible promoter for expression of DARPin-B4	This work / ON681646
005-546-1297-M_B10	DARPin DNA sequence extracted from bacterial expression plasmid bearing an inducible promoter for expression of DARPin-B10	This work / ON681647

005-546-1297-M_B12	DARPin DNA sequence extracted from bacterial expression plasmid bearing an inducible promoter for expression of DARPin-B12	This work / ON681648
005-546-1297-M_C4	DARPin DNA sequence extracted from bacterial expression plasmid bearing an inducible promoter for expression of DARPin-C4	This work / ON681649
005-546-1295-M_C11	DARPin DNA sequence extracted from bacterial expression plasmid bearing an inducible promoter for expression of DARPin-C11	This work / ON681650
005-546-1298-M_D11	DARPin DNA sequence extracted from bacterial expression plasmid bearing an inducible promoter for expression of DARPin-D11	This work / ON681651
005-546-1297-M_D12	DARPin DNA sequence extracted from bacterial expression plasmid bearing an inducible promoter for expression of DARPin-D12	This work / ON681652
005-546-1297-M_E11	DARPin DNA sequence extracted from bacterial expression plasmid bearing an inducible promoter for expression of DARPin-E11	This work / ON681653
005-546-1296-M_F6	DARPin DNA sequence extracted from bacterial expression plasmid bearing an inducible promoter for expression of DARPin-F6	This work / ON681654
005-546-1298-M_F8	DARPin DNA sequence extracted from bacterial expression plasmid bearing an inducible promoter for expression of DARPin-F8	This work / ON681655
005-546-1295-M_F9	DARPin DNA sequence extracted from bacterial expression plasmid bearing an inducible promoter for expression of DARPin-F9	This work / ON681656

005-546-1298-M_F10	DARPin DNA sequence extracted from bacterial expression plasmid bearing an inducible promoter for expression of DARPin-F10	This work / ON681657
005-546-1298-M_F12	DARPin DNA sequence extracted from bacterial expression plasmid bearing an inducible promoter for expression of DARPin-F12	This work / ON681658
005-546-1296-M_G1	Bacterial expression plasmid bearing an inducible promoter for expression of DARPin-G1	This work / ON681660
pAB904	Mammalian expression vector bearing a P_{hCMV}-driven EpoR receptor equipped with 124nc-GFP-DARPin. pMM1 was used as a backbone. (P_{hCMV}-Igk- 124nc-EpoR-IL6st-pA).	This work / ON681661
pAB906	Mammalian expression vector bearing a P_{hCMV}-driven EpoR receptor equipped with R7_5617-GFP_clamp-DARPin. pMM1 was used as a backbone. (P_{hCMV}-Igk- R7_5617-GFP_clamp -EpoR-IL6st-pA).	This work / ON681662
pAB913	Mammalian expression vector bearing a P_{hCMV}-driven EpoR receptor equipped with anti-GFP ReD-nanobody (Kubala, 2010). pMM1 was used as a backbone. (P_{hCMV}-Igk-ReD-EpoR-IL6st-pA).	This work / ON681663
pAB922	Mammalian expression vector bearing a P_{hCMV}-driven EpoR receptor equipped with anti-MBP DARPin (PDB ID: 1SVX) fused to the receptor via a stiff $(EAAAK)_4$ linker. pMM1 was used as a backbone. (P_{hCMV}-Igk-MBP_DARPin-EpoR-IL6st-pA).	This work / ON681664
pAB923	Mammalian expression vector bearing a P_{hCMV}-driven EpoR receptor equipped with anti-MBP scFv (PDB ID: 7JTR_B) fused to the receptor via a stiff $(EAAAK)_4$ linker. pMM1 was used as a backbone. (P_{hCMV}-Igk-MBP_scFv-EpoR-IL6st-pA).	This work / ON681665

pDF101	Inert filler plasmid bearing a bacterial T7 promoter driving an inactive ribozyme (P_{T7}-SpAL-sTRSVac)	Fuchs et al., 2016[1]
pJH6	STAT3-reporter plasmid NanoLuc luciferase reporter expression vector (P_{OSTAT3}-Nluc-pA::P_{hCMV}-STAT3-pA).	This work / ON681666
pLS13	STAT3-reporter plasmid with 2 STAT3 response elements (RE) followed by a minimal promoter driving expression of human secreted placental alkaline phosphatase (SEAP) (RE_2-$P_{hCMVmin}$-SEAP-pA)	Schukur et al. 2015[2] / ON681667
pLS15	Mammalian expression vector bearing the coding sequence of the human STAT3 transcription factor under control of an hCMV promoter (P_{hCMV}-STAT3-pA)	Schukur et al. 2015[2] / ON681668
pMM1	Mammalian expression vector with a modified MCS (P_{hCMV}-MCS-pA; MCS, EcoRI-ATG-SpeI-NheI-BamHI-STOP-XbaI-HindIII-FseI-pA).	Müller et al. 2017[3]
pTS379	Mammalian expression vector bearing a P_{hCMV}-driven EpoR receptor equipped with an anti-D-dimer scFv described by Laroche et al. 1991. pLeo644 (Scheller et al., 2018[4]) was used as a backbone. (P_{hCMV}-Igk-scFv-EpoR-IL6st-pA)	This work / ON681669
pTS380	Mammalian expression vector bearing a P_{hCMV}-driven EpoR receptor equipped with a DARPin as a D-dimer binding moiety. Additional labels indicate the DARPin used as the binding moiety. pLeo644 (Scheller et al., 2018[4]) was used as a backbone (P_{hCMV}-Igk-DARPin-EpoR-IL6st-pA).	This work / ON681670
pTS395	P_{hCMV}-driven Sleeping Beauty transposase mammalian expression vector (P_{hCMV}-SB100-pA).	This work / ON681671

pTS441	STAT3-reporter plasmid with 2 STAT3 response elements (RE) followed by a minimal promoter driving expression of human secreted placental alkaline phosphatase (SEAP) (RE_2-$P_{hCMVmin}$-SEAP-pA) in pMM1 backbone.	This work / ON681672
pTS566	STAT3-reporter plasmid with 4 STAT3 response elements (RE) followed by a minimal promoter driving expression of human secreted placental alkaline phosphatase (SEAP) (RE_2-$P_{hCMVmin}$-SEAP-pA) in pMM1 backbone.	This workScheller et al. 2020[5]
pTS810	STAT3-reporter plasmid with 12 STAT3 response elements (RE) followed by a minimal promoter driving expression of human secreted placental alkaline phosphatase (SEAP) (RE_2-$P_{hCMVmin}$-SEAP-pA) in pMM1 backbone.	This work / ON681673
pTS824	STAT3-reporter plasmid with 16 STAT3 response elements (RE) followed by a minimal promoter driving expression of human secreted placental alkaline phosphatase (SEAP) (RE_2-$P_{hCMVmin}$-SEAP-pA) in pMM1 backbone.	This work / ON681674
pTS835	Stable Sleeping Beauty integration vector bearing three cassettes; an P_{hCMV}-driven EpoR receptor equipped with the anti-D-dimer scFv, an P_{SV40}-driven STAT3 and a P_{RPBSA}-driven selection cassette encoding the blue fluorescent protein mTagBFP2 fused via a p2a peptide sequence to a puromycin resistance gene. (P_{hCMV}-Igk-scFv-EpoR-IL6st-pA-P_{SV40}-STAT3-pA-P_{RPBSA}-mTagBFP2-p2a-PuroR-pA)	This work / ON681675
pTS863	Mammalian expression vector bearing a P_{hCMV}-driven EpoR receptor equipped with DARPin-A7. pMM1 was used as a backbone. (P_{hCMV}-Igk-DARPinA7-EpoR-IL6st-pA)	This work / ON681676

pTS864	Mammalian expression vector bearing a P_{hCMV}-driven EpoR receptor equipped with DARPin-B4. pMM1 was used as a backbone. (P_{hCMV}-Igk-DARPinB4-EpoR-IL6st-pA)	This work / ON681677
pTS865	Mammalian expression vector bearing a P_{hCMV}-driven EpoR receptor equipped with DARPin-G1. pMM1 was used as a backbone. (P_{hCMV}-Igk-DARPinG1-EpoR-IL6st-pA)	This work / ON681678
pTS866	Mammalian expression vector bearing a P_{hCMV}-driven EpoR receptor equipped with a tandem fusion of DARPin-A7 on top of DARPin B4. pMM1 was used as a backbone. (P_{hCMV}-Igk-DARPinA7-DARPinB4-EpoR-IL6st-pA)	This work / ON681679
pTS867	Mammalian expression vector bearing a P_{hCMV}-driven EpoR receptor equipped with a tandem fusion of DARPin-A7 on top of DARPin G1. pMM1 was used as a backbone. (P_{hCMV}-Igk-DARPinA7-DARPinG1-EpoR-IL6st-pA)	This work / ON681680
pTS868	Mammalian expression vector bearing a P_{hCMV}-driven EpoR receptor equipped with a tandem fusion of DARPin-B4 on top of DARPin A7. pMM1 was used as a backbone. (P_{hCMV}-Igk-DARPinB4-DARPinA7-EpoR-IL6st-pA)	This work / ON681681
pTS869	Mammalian expression vector bearing a P_{hCMV}-driven EpoR receptor equipped with a tandem fusion of DARPin-B4 on top of DARPin G1. pMM1 was used as a backbone. (P_{hCMV}-Igk-DARPinB4-DARPinG1-EpoR-IL6st-pA)	This work / ON681682

pTS870	Mammalian expression vector bearing a P_{hCMV}-driven EpoR receptor equipped with a tandem fusion of DARPin-G1 on top of DARPin A7. pMM1 was used as a backbone. (P_{hCMV}-Igk-DARPinG1-DARPinA7-EpoR-IL6st-pA)	This work / ON681683
pTS871	Mammalian expression vector bearing a P_{hCMV}-driven EpoR receptor equipped with a tandem fusion of DARPin-G1 on top of DARPin B4. pMM1 was used as a backbone. (P_{hCMV}-Igk-DARPinG1-DARPinB4-EpoR-IL6st-pA)	This work / ON681684
pTS872	Mammalian expression vector bearing a P_{hCMV}-driven EpoR receptor equipped with a tandem fusion of DARPin-A7 on top of DARPin A7. pMM1 was used as a backbone. (P_{hCMV}-Igk-DARPinA7-DARPinA7-EpoR-IL6st-pA)	This work / ON681685
pTS873	Mammalian expression vector bearing a P_{hCMV}-driven EpoR receptor equipped with a tandem fusion of DARPin-B4 on top of DARPin B4. pMM1 was used as a backbone. (P_{hCMV}-Igk-DARPinB4-DARPinB4-EpoR-IL6st-pA)	This work / ON681686
pTS874	Mammalian expression vector bearing a P_{hCMV}-driven EpoR receptor equipped with a tandem fusion of DARPin-G1 on top of DARPin G1. pMM1 was used as a backbone. (P_{hCMV}-Igk-DARPinG1-DARPinG1-EpoR-IL6st-pA)	This work / ON681687
pTS914	Stable Sleeping Beauty integration vector bearing two cassettes; a P_{hCMV}-driven EpoR receptor equipped with DARPin B4 and a P_{RPBSA}-driven selection cassette encoding a selection marker for blasticidin resistance. (PhCMV-Igk-DARPinB4-EpoR-IL6st-pA- P_{RPBSA}-BlastR-pA)	This work / ON681688

pTS922	Mammalian expression vector bearing a P_{hCMV}-driven EpoR receptor equipped with the anti-D-dimer scFv. pMM1 was used as a backbone. (P_{hCMV}-Igk-scFv-EpoR-IL6st-pA)	This work / ON681689
pTS930	Mammalian expression vector bearing a P_{hCMV}-driven EpoR receptor equipped with a tandem fusion of the anti-D-dimer scFv on top of DARPin-A7. pMM1 was used as a backbone. (P_{hCMV}-Igk-scFv-DARPinA7-EpoR-IL6st-pA)	This work / ON681690
pTS931	Mammalian expression vector bearing a P_{hCMV}-driven EpoR receptor equipped with a tandem fusion of the anti-D-dimer scFv on top of DARPin-B4. pMM1 was used as a backbone. (P_{hCMV}-Igk-scFv-DARPinB4-EpoR-IL6st-pA)	This work / ON681691
pTS932	Mammalian expression vector bearing a P_{hCMV}-driven EpoR receptor equipped with a tandem fusion of the anti-D-dimer scFv on top of DARPin-G1. pMM1 was used as a backbone. (P_{hCMV}-Igk-scFv-DARPinG1-EpoR-IL6st-pA)	This work / ON681692
pTS941	Stable Sleeping Beauty integration vector bearing three cassettes; an P_{hCMV}-driven EpoR receptor equipped with a tandem fusion of the anti-D-dimer scFv on top of DARPin-B4, an P_{SV40}-driven STAT3 and a RPBSA-driven selection cassette encoding the blue fluorescent protein mTagBFP2 fused via a p2a peptide sequence to a selection marker for puromycin resistance. (P_{hCMV}-Igk-scFv-DARPinB4-EpoR-IL6st-pA-P_{SV40}-STAT3-pA-P_{PRBSA}-mTagBFP2-p2a-PuroR-pA)	This work / ON681693
pTS942	Stable Sleeping Beauty integration vector bearing three cassettes; an P_{hCMV}-driven EpoR receptor equipped with an scFv against the industrial dye reactive red (RR120) as described in Scheller et al	This work / ON681694

	2018[4], an P_{SV40}-driven STAT3 and a P_{RPBSA}-driven selection cassette encoding the blue fluorescent protein mTagBFP2 fused via a p2a peptide sequence to a selection marker for puromycin resistance. (P_{hCMV}-Igk-scFv(RR120)-EpoR-IL6st-pA-P_{SV40}-STAT3-pA-P_{RPBSA}-mTagBFP2-p2a-PuroR-pA)	
pTS992	Stable Sleeping Beauty integration vector bearing two cassettes; four repeats of the STAT3 response element sequence followed by a minimal promoter driving expression of a secreted nano-luciferase fused to a murine Fc (mFc) tag separated by a cleavage site for furin from an also Fc-stabilized copy of tenecteplase (TNK). A second cassette drives expression of a selection marker for zeocin resistance fused to the yellow fluorescent protein YPet via a p2a peptide sequence. (RE_4-$P_{hCMVmin}$-Igk-Nluc-mFc-Furin-TNK-mFc-pA-PRBSA-ZeoR-p2a-YPet-pA)	This work / ON681696
pTS2011	Mammalian expression vector bearing a P_{hCMV}-driven EpoR receptor equipped with an scFv against the industrial dye reactive red (RR120) as described in Scheller et al 2018[4]. This plasmid was used as a negative control. pMM1 was used as a backbone. (P_{hCMV}-Igk-scFv(RR120)-EpoR-IL6st-pA)	This work / ON681697
pTS2151	Stable Sleeping Beauty integration vector bearing two cassettes; four repeats of the STAT3 operator sequence followed by a minimal promoter driving expression of a secreted nano-luciferase fused to a murine Fc (mFc) tag separated by a p2a site that separates translation of Nluc and hirudin-HM2. A second cassette drives expression of a selection marker for zeocin resistance fused to the yellow fluorescent protein YPet via a p2a peptide sequence. (RE_4-$P_{hCMVmin}$-Igk-Nluc-mFc-p2a-HIRM2-pA-PRBSA-ZeoR-p2a-YPet-pA)	This work / ON681698

pTS2165	Mammalian expression vector bearing a P_{hCMV}-driven EpoR receptor equipped with a double tandem fusion of the anti-D-dimer scFv. pMM1 was used as a backbone.<br>(P_{hCMV}-D-dimer-scFv-D-dimer-scFv-receptor-pA)	This work / ON681699
pTS2166	Mammalian expression vector bearing a P_{hCMV}-driven EpoR receptor equipped with a tandem fusion of DARPin-A7 on top of the anti-D-dimer scFv. pMM1 was used as a backbone.<br>(P_{hCMV}-DARPinA7-D-dimer-scFv-receptor-pA)	This work / ON681700
pTS2167	Mammalian expression vector bearing a P_{hCMV}-driven EpoR receptor equipped with a tandem fusion of DARPin-B4 on top of the anti-D-dimer scFv. pMM1 was used as a backbone.<br>(P_{hCMV}-DARPinB4-D-dimer-scFv(tandem)-receptor-pA)	This work / ON681701
pTS2168	Mammalian expression vector bearing a P_{hCMV}-driven EpoR receptor equipped with a tandem fusion of DARPin-G1on top of the anti-D-dimer scFv. pMM1 was used as a backbone.<br>(P_{hCMV}-DARPinG1-D-dimer-scFv(tandem)-receptor-pA)	This work / ON681702

Supplementary Table 2: Plasmids transfected in each experiment

Details of the transfection protocol can be found in the methods.

Figure	Plasmids used
1<br>c)	2 ng of pAB906: P_{hCMV}-GFP_Clamp_DARPin-receptor-pA<br>30 ng of each pLS13 and pLS15 used to build the reporter system,<br>pDF101 to adjust plasmid DNA amount to 200 ng per transfection
1<br>d)	2 ng of pAB904: P_{hCMV}-GFP_124nc_DARPin-receptor-pA<br>30 ng of each pLS13 and pLS15 used to build the reporter system,<br>pDF101 to adjust plasmid DNA amount to 200 ng per transfection
1<br>e)	2 ng of pAB913: P_{hCMV}-GFP_nanobody-receptor-pA<br>30 ng of each pLS13 and pLS15 used to build the reporter system,<br>pDF101 to adjust plasmid DNA amount to 200 ng per transfection
1<br>f)	pAB922: P_{hCMV}-MBP_DARPin-(EAAAK)$_4$-receptor-pA<br>pAB923: P_{hCMV}-MBP_scFv-(EAAAK)$_4$-receptor-pA<br>2 ng of each of the indicated receptor plasmids,<br>30 ng of each pLS13 and pLS15 used to build the reporter system,<br>pDF101 to adjust plasmid DNA amount to 200 ng per transfection
1 h)	Plasmid library for loop DARPins

2<br><br>b)	pTS380-A12: P_{hCMV}-DARPinA12-receptor-pA<br>pTS380-B4: P_{hCMV}-DARPinB4-receptor-pA<br>pTS380-D11: P_{hCMV}-DARPinD11-receptor-pA<br>pTS380-C11: P_{hCMV}-DARPinC11-receptor-pA<br>pTS380-F12: P_{hCMV}-DARPinF12-receptor-pA<br>pTS380-A10: P_{hCMV}-DARPinA10-receptor-pA<br>pTS380-D12: P_{hCMV}-DARPinD12-receptor-pA<br>pTS380-F10: P_{hCMV}-DARPinF10-receptor-pA<br>pTS380-A6: P_{hCMV}-DARPinA6-receptor-pA<br>pTS380-B12: P_{hCMV}-DARPinB12-receptor-pA<br>pTS380-F8: P_{hCMV}-DARPinF8-receptor-pA<br>pTS380-B10: P_{hCMV}-DARPinB10-receptor-pA<br>pTS380-F9: P_{hCMV}-DARPinF9-receptor-pA<br>pTS380-A7: P_{hCMV}-DARPinA7-receptor-pA<br>pTS380-A11: P_{hCMV}-DARPinA11-receptor-pA<br>pTS380-C4: P_{hCMV}-DARPinC4-receptor-pA<br>pTS380-F6: P_{hCMV}-DARPinF6-receptor-pA<br>pTS380-E11: P_{hCMV}-DARPinE11-receptor-pA

	pTS380-G1: P_{hCMV}-DARPinG1-receptor-pA<br>pTS379: P_{hCMV}-D-dimer-scFv-receptor-pA<br>pTS2011: P_{hCMV}-RR120 -scFv-receptor-pA<br>2 ng of the indicated receptor plasmids,<br>30 ng of each pLS13 and pLS15 used to build the reporter system,<br>pDF101 to adjust plasmid DNA amount to 200 ng per transfection
2<br>c) – d)<br>f) – g)	pTS380-A6: P_{hCMV}-DARPinA6-receptor-pA<br>pTS380-A7: P_{hCMV}-DARPinA7-receptor-pA<br>pTS380-B4: P_{hCMV}-DARPinB4-receptor-pA<br>pTS380-G1: P_{hCMV}-DARPinG1-receptor-pA<br>pTS379: P_{hCMV}-D-dimer-scFv-receptor-pA<br>pTS2011: P_{hCMV}-RR120 -scFv-receptor-pA<br>2 ng of the indicated receptor plasmids,<br>30 ng of each pLS13 and pLS15 used to build the reporter system,<br>pDF101 to adjust plasmid DNA amount to 200 ng per transfection
2<br>e)<br>h)	pTS870: P_{hCMV}-DARPinG1-A7(tandem)-receptor-pA<br>pTS871: P_{hCMV}-DARPinG1-B4(tandem)-receptor-pA<br>pTS872: P_{hCMV}-DARPinG1-G1(tandem)-receptor-pA

	pTS930: P_{hCMV}-D-dimer-scFv-DARPinA7(tandem)-receptor-pA<br>pTS931: P_{hCMV}-D-dimer-scFv-DARPinB4(tandem)-receptor-pA<br>pTS932: P_{hCMV}-D-dimer-scFv-DARPinG1(tandem)-receptor-pA<br>pTS2011: P_{hCMV}-RR120-scFv-receptor-pA<br>2 ng of the indicated receptor plasmids,<br>30 ng of each pLS13 and pLS15 used to build the reporter system,<br>pDF101 to adjust plasmid DNA amount to 200 ng per transfection
3<br>a)-c)	pTS864: P_{hCMV}-DARPinB4-receptor-pA<br>pTS871: P_{hCMV}-DARPinG1-B4(tandem)-receptor-pA<br>pTS922: P_{hCMV}-D-dimer-scFv-receptor-pA<br>pTS931: P_{hCMV}-D-dimer-scFv-DARPinB4(tandem)-receptor-pA<br>2 ng of the indicated receptor plasmids,<br>30 ng of each pLS13 and pLS15 used to build the reporter system,<br>pDF101 to adjust plasmid DNA amount to 200 ng per transfection
4<br>a)	pTS931: P_{hCMV}-D-dimer-scFv-DARPinB4(tandem)-receptor-pA<br>pTS2011: P_{hCMV}-RR120-scFv-receptor-pA<br>2 ng of the indicated receptor plasmids,<br>30 ng of each pLS13 and pLS15 used to build the reporter system,

	pDF101 to adjust plasmid DNA amount to 200 ng per transfection
4<br>b)	pTS864: P_{hCMV}-DARPinB4-receptor-pA<br>pTS922: P_{hCMV}-D-dimer-scFv-receptor-pA 2 ng of the indicated receptor plasmids,<br>30 ng of each pLS13 and pLS15 used to build the reporter system,<br>pDF101 to adjust plasmid DNA amount to 200 ng per transfection
4<br>c)	Stable cell line $HEK_{scFv-B4/TNK}$ was generated using the following plasmids<br>pTS941: P_{hCMV}-D-dimer-scFv-DARPinB4(tandem)-receptor-pA-P_{SV40}-STAT3-pA-P_{hCMV}-TagBFP2-p2a-PuroR-pA<br>pcTS992: RE_4-P_{hCMV}-IgkSS-Nluc- mF_c-RARYKR-TNK- mF_c-P_{hCMV}-ZeoR-p2a-YPet-pA<br>pcTS395: P_{hCMV}-SleepingBeauty-transposase-pA<br>pDF101 to adjust plasmid DNA amount to 1 µg per transfection
4<br>e) – f)	Stable cell line $mHEK_{B4/scFv/hirudin}$ was generated using the following plasmids<br>pTS835: P_{hCMV}-D-dimer-scFv-receptor-pA-P_{SV40}-STAT3-pA-P_{hCMV}-TagBFP2-p2a-PuroR-pA<br>pTS914: P_{hCMV}-DARPinB4-receptor-pA-P_{hCMV}-BlastR-pA<br>pTS2151: RE_4-P_{hCMV}-IgkSS-Nluc-mF_c-p2a-HIRM2-pA-P_{hCMV}-ZeoR-p2a-YPet-pA<br>pcTS395: P_{hCMV}-SleepingBeauty-transposase-pA<br>pDF101 to adjust plasmid DNA amount to 1 µg per transfection
4<br>h), j)	pTS864: P_{hCMV}-DARPinB4-EpoR-pA), 30 µg<br>pTS922 (P_{hCMV}-scFv-EpoR-pA), 30 µg

	pJH6 (P_{OSTAT3}-NLuc-pA::P_{hCMV}-STAT3-pA), 300 µg<br>Plasmids were hydrodynamically injected into each mouse via the tail vein within 3-5 seconds.
S3	Plasmid library for loop DARPins
S4<br>a)	pTS380-B4: P_{hCMV}-DARPinB4-receptor-pA<br>pTS864: P_{hCMV}-DARPinB4-receptor-pA<br>pTS379: P_{hCMV}-D-dimer-scFv-receptor-pA<br>pLS13 and pLS15 used to build the reporter system<br>pDF101 to adjust plasmid DNA amount to 200 ng per transfection
S4<br>b)	pLeo615: P_{hCMV}-RR120-scFv-receptor-pA<br>pLS13 and pLS15 used to build the reporter system<br>pDF101 to adjust plasmid DNA amount to 200 ng per transfection
S4<br>c)<br>e)	100 ng of each of the following plasmids was used per transfection<br>pSEAP2ctr: P_{SV40}-SEAP-pA<br>pFS29: P_{SV40}-mCherry-pA
S5<br>a) – d)	pTS380-A12: P_{hCMV}-DARPinA12-receptor-pA<br>pTS380-B4: P_{hCMV}-DARPinB4-receptor-pA<br>pTS380-D11: P_{hCMV}-DARPinD11-receptor-pA<br>pTS380-C11: P_{hCMV}-DARPinC11-receptor-pA

	pTS380-F12: P_{hCMV}-DARPinF12-receptor-pA<br>pTS380-A10: P_{hCMV}-DARPinA10-receptor-pA<br>pTS380-D12: P_{hCMV}-DARPinD12-receptor-pA<br>pTS380-F10: P_{hCMV}-DARPinF10-receptor-pA<br>pTS380-A6: P_{hCMV}-DARPinA6-receptor-pA<br>pTS380-B12: P_{hCMV}-DARPinB12-receptor-pA<br>pTS380-F8: P_{hCMV}-DARPinF8-receptor-pA<br>pTS380-B10: P_{hCMV}-DARPinB10-receptor-pA<br>pTS380-F9: P_{hCMV}-DARPinF9-receptor-pA<br>pTS380-A7: P_{hCMV}-DARPinA7-receptor-pA<br>pTS380-A11: P_{hCMV}-DARPinA11-receptor-pA<br>pTS380-C4: P_{hCMV}-DARPinC4-receptor-pA<br>pTS380-F6: P_{hCMV}-DARPinF6-receptor-pA<br>pTS380-E11: P_{hCMV}-DARPinE11-receptor-pA<br>pTS380-G1: P_{hCMV}-DARPinG1-receptor-pA<br>pTS379: P_{hCMV}-D-dimer-scFv-receptor-pA<br>pTS2011: P_{hCMV}-RR120 -scFv-receptor-pA<br>pLS13 and pLS15 used to build the reporter system

	pDF101 to adjust plasmid DNA amount to 200 ng per transfection
S7<br>a)<br>b)	pTS866: P_{hCMV}-DARPinA7-B4(tandem)-receptor-pA<br>pTS867: P_{hCMV}-DARPinA7-G1 (tandem)-receptor-pA<br>pTS868: P_{hCMV}-DARPinB4-A7(tandem)-receptor-pA<br>pTS869: P_{hCMV}-DARPinB4-G1(tandem)-receptor-pA<br>pTS870: P_{hCMV}-DARPinG1-A7(tandem)-receptor-pA<br>pTS871: P_{hCMV}-DARPinG1-B4(tandem)-receptor-pA<br>pTS872: P_{hCMV}-DARPinA7-A7(tandem)-receptor-pA<br>pTS873: P_{hCMV}-DARPinB4-B4(tandem)-receptor-pA<br>pTS874: P_{hCMV}-DARPinG1-G1(tandem)-receptor-pA<br>pTS2011: P_{hCMV}-RR120-scFv-receptor-pA<br>pLS13 and pLS15 used to build the reporter system<br>pDF101 to adjust plasmid DNA amount to 200 ng per transfection
S7<br>c)<br>d)	pTS922: P_{hCMV}-D-dimer-scFv-receptor-pA<br>pTS2166: P_{hCMV}-DARPinA7-D-dimer-scFv(tandem)-receptor-pA<br>pTS2167: P_{hCMV}-DARPinB4-D-dimer-scFv(tandem)-receptor-pA<br>pTS2168: P_{hCMV}-DARPinG1-D-dimer-scFv(tandem)-receptor-pA<br>pTS930: P_{hCMV}-D-dimer-scFv-DARPinA7(tandem)-receptor-pA

	pTS931: P_{hCMV}-D-dimer-scFv-DARPinB4(tandem)-receptor-pA<br>pTS932: P_{hCMV}-D-dimer-scFv-DARPinG1(tandem)-receptor-pA<br>pTS2165: P_{hCMV}-D-dimer-scFv-D-dimer-scFv(tandem)-receptor-pA<br>pTS2011: P_{hCMV}-RR120-scFv-receptor-pA<br>pLS13 and pLS15 used to build the reporter system<br>pDF101 to adjust plasmid DNA amount to 200 ng per transfection
S9<br>a),b), d)	pTS380-A6: P_{hCMV}-DARPinA6-receptor-pA<br>pTS380-A7: P_{hCMV}-DARPinA7-receptor-pA<br>pTS380-B4: P_{hCMV}-DARPinB4-receptor-pA<br>pTS380-G1: P_{hCMV}-DARPinG1-receptor-pA<br>pTS379: P_{hCMV}-D-dimer-scFv-receptor-pA<br>pTS2011: P_{hCMV}-RR120 -scFv-receptor-pA<br>25 ng of the indicated receptor plasmids,<br>pDF101 to adjust plasmid DNA amount to 250 ng per transfection in a 24-well plate.
S10<br>a)-c)	pTS864: P_{hCMV}-DARPinB4-receptor-pA<br>pTS871: P_{hCMV}-DARPinG1-B4(tandem)-receptor-pA<br>pTS922: P_{hCMV}-D-dimer-scFv-receptor-pA<br>pTS931: P_{hCMV}-D-dimer-scFv-DARPinB4(tandem)-receptor-pA

	pTS2011: P_{hCMV}-RR120-scFv-receptor-pA<br>pLS13 and pLS15 used to build the reporter system<br>pDF101 to adjust plasmid DNA amount to 200 ng per transfection
S11<br>a)-c)	pTS864: P_{hCMV}-DARPinB4-receptor-pA<br>pTS922: P_{hCMV}-D-dimer-scFv-receptor-pA<br>pTS2011: P_{hCMV}-RR120-scFv-receptor-pA<br>pLS13 and pLS15 used to build the reporter system<br>pDF101 to adjust plasmid DNA amount to 200 ng per transfection
S12<br>a)	pTS380-B4: P_{hCMV}-DARPinB4-receptor-pA<br>pTS379: P_{hCMV}-D-dimer-scFv-receptor-pA<br>pLS13 and pLS15 used to build the reporter system<br>pDF101 to adjust plasmid DNA amount to 200 ng per transfection
S13<br>a)	pTS2011: P_{hCMV}-RR120-scFv-receptor-pA<br>pLS13 and pLS15 used to build the reporter system<br><br>pSEAP2control: P_{SV40}-SEAP-pA<br>pDF101 to adjust plasmid DNA amount to 200 ng per transfection
S13	pTS864: P_{hCMV}-DARPinB4-receptor-pA

b-c)	pTS922: P_{hCMV}-D-dimer-scFv-receptor-pA<br>pTS871: P_{hCMV}-DARPinG1-B4(tandem)-receptor-pA<br>pTS2011: P_{hCMV}-RR120-scFv-receptor-pA<br>pLS13 and pLS15 used to build the reporter system<br>pDF101 to adjust plasmid DNA amount to 200 ng per transfection
S13<br>d-e)	pTS864: P_{hCMV}-DARPinB4-receptor-pA<br>pTS922: P_{hCMV}-D-dimer-scFv-receptor-pA<br>pTS871: P_{hCMV}-DARPinG1-B4(tandem)-receptor-pA<br>pTS931: P_{hCMV}-D-dimer-scFv-DARPinB4(tandem)-receptor-pA<br>pTS2011: P_{hCMV}-RR120-scFv-receptor-pA<br>pLS13 and pLS15 used to build the reporter system<br><br>pSEAP2control: P_{SV40}-SEAP-pA<br>pDF101 to adjust plasmid DNA amount to 200 ng per transfection
S14	pTS2011: P_{hCMV}-RR120-scFv-receptor-pA<br>pLS13 and pLS15 used to build the reporter system<br><br>pSEAP2control: P_{SV40}-SEAP-pA

	pDF101 to adjust plasmid DNA amount to 200 ng per transfection
S15<br>a)	pTS380-B4: P_{hCMV}-DARPinB4-receptor-pA<br>pTS379: P_{hCMV}-D-dimer-scFv-receptor-pA<br>pTS539: P_{SV40}-STAT3-pA<br>pLS13: RE_{2}-$p_{hCMVmin}$-SEAP-pA<br>pTS441: RE_{2}-$p_{hCMVmin}$-SEAP-pA<br>pTS566: RE_{4}-$p_{hCMVmin}$-SEAP-pA<br>pTS810: RE_{12}-$p_{hCMVmin}$-SEAP-pA<br>pTS824: RE_{16}-$p_{hCMVmin}$-SEAP-pA<br>pDF101 to adjust plasmid DNA amount to 200 ng per transfection
S15<br>b-f)	Stable cell lines $HEK_{scFv-B4/TNK}$ and $HEK_{RR120/TNK}$ were generated using the following plasmids<br><br>$HEK_{scFv-B4/TNK}$:<br>pTS941: P_{hCMV}-D-dimer-scFv-DARPinB4(tandem)-receptor-pA-P_{SV40}-STAT3-pA-P_{hCMV}-TagBFP2-p2a-PuroR-pA<br>pcTS992: RE_{4}-P_{hCMV}-IgkSS-Nluc-mF_{c}-RARYKR-TNK-mF_{c}-P_{hCMV}-ZeoR-p2a-YPet-pA<br><br>$HEK_{RR120/TNK}$:<br>pTS942: P_{hCMV}-RR120-scFv-receptor-pA-P_{SV40}-STAT3-pA-P_{hCMV}-TagBFP2-p2a-PuroR-pA

	pcTS992: RE_4-P_{hCMV}-IgkSS-Nluc-mF_c-RARYKR-TNK-mF_c-P_{hCMV}-ZeoR-p2a-YPet-pA<br>pcTS395: P_{hCMV}-SleepingBeauty-transposase-pA<br>pDF101 to adjust plasmid DNA amount to 1 µg per transfection
S17	pTS380-A6: P_{hCMV}-DARPinA6-receptor-pA<br>pTS380-A7: P_{hCMV}-DARPinA7-receptor-pA<br>pTS380-B4: P_{hCMV}-DARPinB4-receptor-pA<br>pTS380-G1: P_{hCMV}-DARPinG1-receptor-pA<br>2 ng of the indicated receptor plasmids,<br>30 ng of each pLS13 and pLS15 used to build the reporter system,<br>pDF101 to adjust plasmid DNA amount to 200 ng per transfection

Supplementary Table 3: Amino acid sequences of GFP-GFP fusions

Details of the purification can be found in the methods. Linker sequences and fusion sites are highlighted in bold and underlined.

Construct	Sequence (AA)
Long linker ($GGGGS_4$)	GSMVSKGEELFTGVVPILVELDGDVNGHKFSVSGEGEGDATYGKLTLKFICTTGKLPVPW<br>PTLVTTLTYGVQCFSRYPDHMKQHDFFKSAMPEGYVQERTIFFKDDGNYKTRAEVKFEGD<br>TLVNRIELKGIDFKEDGNILGHKLEYNYNSHNVYIMADKQKNGIKVNFKIRHNIEDGSVQ<br>LADHYQQNTPIGDGPVLLPDNHYLSTQSALSKDPNEKRDHMVLLEFVTAAGITLGMDELY<br>K**<u>GGGGSGGGGSGGGGSGGGGS</u>**MVSKGEELFTGVVPILVELDGDVNGHKFSVSGEGEGDAT<br>YGKLTLKFICTTGKLPVPWPTLVTTLTYGVQCFSRYPDHMKQHDFFKSAMPEGYVQERTI<br>FFKDDGNYKTRAEVKFEGDTLVNRIELKGIDFKEDGNILGHKLEYNYNSHNVYIMADKQK<br>NGIKVNFKIRHNIEDGSVQLADHYQQNTPIGDGPVLLPDNHYLSTQSALSKDPNEKRDHM<br>VLLEFVTAAGITLGMDELYK
Short linker (GGGGS**)**	GSMVSKGEELFTGVVPILVELDGDVNGHKFSVSGEGEGDATYGKLTLKFICTTGKLPVPW<br>PTLVTTLTYGVQCFSRYPDHMKQHDFFKSAMPEGYVQERTIFFKDDGNYKTRAEVKFEGD<br>TLVNRIELKGIDFKEDGNILGHKLEYNYNSHNVYIMADKQKNGIKVNFKIRHNIEDGSVQ<br>LADHYQQNTPIGDGPVLLPDNHYLSTQSALSKDPNEKRDHMVLLEFVTAAGITLGMDELY<br>K**<u>GGGGS</u>**MVSKGEELFTGVVPILVELDGDVNGHKFSVSGEGEGDATYGKLTLKFICTTGKL<br>PVPWPTLVTTLTYGVQCFSRYPDHMKQHDFFKSAMPEGYVQERTIFFKDDGNYKTRAEVK<br>FEGDTLVNRIELKGIDFKEDGNILGHKLEYNYNSHNVYIMADKQKNGIKVNFKIRHNIED<br>GSVQLADHYQQNTPIGDGPVLLPDNHYLSTQSALSKDPNEKRDHMVLLEFVTAAGITLGM<br>DELYK
No linker	GSMVSKGEELFTGVVPILVELDGDVNGHKFSVSGEGEGDATYGKLTLKFICTTGKLPVPW<br>PTLVTTLTYGVQCFSRYPDHMKQHDFFKSAMPEGYVQERTIFFKDDGNYKTRAEVKFEGD<br>TLVNRIELKGIDFKEDGNILGHKLEYNYNSHNVYIMADKQKNGIKVNFKIRHNIEDGSVQ<br>LADHYQQNTPIGDGPVLLPDNHYLSTQSALSKDPNEKRDHMVLLEFVTAAGIT**<u>LG</u>**MVSKG<br>EELFTGVVPILVELDGDVNGHKFSVSGEGEGDATYGKLTLKFICTTGKLPVPWPTLVTTL<br>TYGVQCFSRYPDHMKQHDFFKSAMPEGYVQERTIFFKDDGNYKTRAEVKFEGDTLVNRIE<br>LKGIDFKEDGNILGHKLEYNYNSHNVYIMADKQKNGIKVNFKIRHNIEDGSVQLADHYQQ<br>NTPIGDGPVLLPDNHYLSTQSALSKDPNEKRDHMVLLEFVTAAGITLGMDELYK
Monomeric	GSMVSKGEELFTGVVPILVELDGDVNGHKFSVSGEGEGDATYGKLTLKFICTTGKLPVPW<br>PTLVTTLTYGVQCFSRYPDHMKQHDFFKSAMPEGYVQERTIFFKDDGNYKTRAEVKFEGD<br>TLVNRIELKGIDFKEDGNILGHKLEYNYNSHNVYIMADKQKNGIKVNFKIRHNIEDGSVQ<br>LADHYQQNTPIGDGPVLLPDNHYLSTQSALSKDPNEKRDHMVLLEFVTAAGITLGMDELY<br>K

Additional References

1 Auslander, S., Fuchs, D., Hurlemann, S., Auslander, D. & Fussenegger, M. Engineering a ribozyme cleavage-induced split fluorescent aptamer complementation assay. *Nucleic Acids Res* **44**, e94, doi:10.1093/nar/gkw117 (2016).

2 Schukur, L., Geering, B., Charpin-El Hamri, G. & Fussenegger, M. Implantable synthetic cytokine converter cells with AND-gate logic treat experimental psoriasis. *Sci Transl Med* **7**, 318ra201, doi:10.1126/scitranslmed.aac4964 (2015).

3 Muller, M. *et al.* Designed cell consortia as fragrance-programmable analog-to-digital converters. *Nat Chem Biol* **13**, 309-316, doi:10.1038/nchembio.2281 (2017).

4 Scheller, L., Strittmatter, T., Fuchs, D., Bojar, D. & Fussenegger, M. Generalized extracellular molecule sensor platform for programming cellular behavior. *Nat Chem Biol* **14**, 723-+, doi:10.1038/s41589-018-0046-z (2018).

5 Scheller L, Schmollack M, Bertschi A, Mansouri M, Saxena P, Fussenegger M. Phosphoregulated orthogonal signal transduction in mammalian cells. Nature Communications 2020; 11. doi:10.1038/s41467-020-16895-1.
