# Supplementary material for: Programmable DARPin-based receptors for the detection of thrombotic markers
Source: Nat Chem Biol. 2022 Aug 8;18(10):1125–34. doi: 10.1038/s41589-022-01095-3 (PMC9512699; doi:10.1038/s41589-022-01095-3)
Supplement: Supplementary file 2 — Reporting Summary [file 41589_2022_1095_MOESM2_ESM.pdf]

nature research

Corresponding author(s): Martin Fussenegger

Last updated by author(s): Jun 26, 2021

Reporting Summary

Nature Research wishes to improve the reproducibility of the work that we publish. This form provides structure for consistency and transparency in reporting. For further information on Nature Research policies, see our Editorial Policies and the Editorial Policy Checklist.

Statistics

For all statistical analyses, confirm that the following items are present in the figure legend, table legend, main text, or Methods section.

n/a	Confirmed	
☐	☒	The exact sample size (*n*) for each experimental group/condition, given as a discrete number and unit of measurement
☐	☒	A statement on whether measurements were taken from distinct samples or whether the same sample was measured repeatedly
☐	☒	The statistical test(s) used AND whether they are one- or two-sided<br>*Only common tests should be described solely by name; describe more complex techniques in the Methods section.*
☒	☐	A description of all covariates tested
☒	☐	A description of any assumptions or corrections, such as tests of normality and adjustment for multiple comparisons
☐	☒	A full description of the statistical parameters including central tendency (e.g. means) or other basic estimates (e.g. regression coefficient) AND variation (e.g. standard deviation) or associated estimates of uncertainty (e.g. confidence intervals)
☐	☒	For null hypothesis testing, the test statistic (e.g. *F*, *t*, *r*) with confidence intervals, effect sizes, degrees of freedom and *P* value noted<br>*Give P values as exact values whenever suitable.*
☒	☐	For Bayesian analysis, information on the choice of priors and Markov chain Monte Carlo settings
☒	☐	For hierarchical and complex designs, identification of the appropriate level for tests and full reporting of outcomes
☒	☐	Estimates of effect sizes (e.g. Cohen's *d*, Pearson's *r*), indicating how they were calculated

Our web collection on statistics for biologists contains articles on many of the points above.

Software and code

Policy information about availability of computer code

Data collection	Microsoft Excel for Mac version 16.50
Data analysis	GraphPad Prism for MacOS version 9.1

For manuscripts utilizing custom algorithms or software that are central to the research but not yet described in published literature, software must be made available to editors and reviewers. We strongly encourage code deposition in a community repository (e.g. GitHub). See the Nature Research guidelines for submitting code & software for further information.

Data

Policy information about availability of data

All manuscripts must include a data availability statement. This statement should provide the following information, where applicable:

- Accession codes, unique identifiers, or web links for publicly available datasets
- A list of figures that have associated raw data
- A description of any restrictions on data availability

All data measured and analyzed in this study is available from the corresponding author on reasonable request.

Field-specific reporting

Please select the one below that is the best fit for your research. If you are not sure, read the appropriate sections before making your selection.

☒ Life sciences ☐ Behavioural & social sciences ☐ Ecological, evolutionary & environmental sciences

For a reference copy of the document with all sections, see nature.com/documents/nr-reporting-summary-flat.pdf

Life sciences study design

All studies must disclose on these points even when the disclosure is negative.

Sample size	No sample size calculation was done. Sample size (n=3 independent biological samples) was predicted to be sufficient to estimate the variation of the data and hence differences in means based on previous experiences on reporter gene expression
Data exclusions	No data points were excluded.
Replication	All in vitro data was successfully replicated. Data from mouse experiments was not replicated due to sufficient group size and ethical reasons.
Randomization	No covariates due to sample allocation could be observed in cell culture and no randomization was performed. Experiments were done using the same mastermix to transfect all conditions of the same type. Treatments were also performed using the same solution to induce respective samples of mock/negative controls and functional receptor configurations. Differently transfected cells were all cultured in the same 96-well plate where the outer wells were left out to account for potential edge effects.<br>In mouse experiments, mice were randomly selected for each group.
Blinding	Cell culture experiments were not blinded since the use of multichannel pipetting and highly parallelized workflows for samples and controls reduce the likelihood to introduce even subconcious biases.<br>The blood sample analysis from mouse experiments was blinded.

Reporting for specific materials, systems and methods

We require information from authors about some types of materials, experimental systems and methods used in many studies. Here, indicate whether each material, system or method listed is relevant to your study. If you are not sure if a list item applies to your research, read the appropriate section before selecting a response.

Materials & experimental systems

n/a	Involved in the study	
☒	☐	Antibodies
☐	☒	Eukaryotic cell lines
☒	☐	Palaeontology and archaeology
☐	☒	Animals and other organisms
☐	☒	Human research participants
☒	☐	Clinical data
☒	☐	Dual use research of concern

Methods

n/a	Involved in the study	
☒	☐	ChIP-seq
☒	☐	Flow cytometry
☒	☐	MRI-based neuroimaging

Eukaryotic cell lines

Policy information about cell lines

Cell line source(s)	HEK-293T (ACC 635) were purchased from the German Collection of Microorganisms and Cell Cultures (DSMZ).
Authentication	Cells were recently obtained from the cell bank (DSMZ) or thawed from certified stocks and no further confirmation was performed
Mycoplasma contamination	Cells were aquired mycoplasma free from ATCC and cultured under antibiotic free conditions, no test for mycoplasma contamination was performed.
Commonly misidentified lines (See ICLAC register)	Cells used are not listed in the ICLAC register.

Animals and other organisms

Policy information about studies involving animals; ARRIVE guidelines recommended for reporting animal research

Laboratory animals	Wild-type male 6-week-old BABL/c mice were used in this study.

Wild animals	This study did not involve wild animals.
Field-collected samples	No samples were collected in the field.
Ethics oversight	The experiments involving animals were approved by the East China Normal University (ECNU) Animal Care and Use Committee and were in accordance with the Ministry of Science and Technology of the People's Republic of China guidelines on animal care.

Note that full information on the approval of the study protocol must also be provided in the manuscript.

Human research participants

Policy information about studies involving human research participants

Population characteristics	Blood samples were collected from routine blood testing at the hospital. Patients with DIC and regular patients were included. No additional information was obtained due to blinding.
Recruitment	Patient samples were collected from routine blood testing at the hospital. Samples showing normal and high D-dimer levels were selected.
Ethics oversight	Experiments involving patient samples were approved by the Medical Ethics Committee of Yangpu Hospital affiliated with Tongji University (request ID: LL-2018-SCI-003) and the Ethikkommission Nordwest- und Zentralschweiz (EKNZ) (request ID: 2021-01998).

Note that full information on the approval of the study protocol must also be provided in the manuscript.
